# Cannabinoids, Endocannabinoids and Sleep

**DOI:** 10.3389/fnmol.2020.00125

**Published:** 2020-07-22

**Authors:** Andrew J. Kesner, David M. Lovinger

**Affiliations:** ^1^Division of Intramural Clinical and Biological Research, National Institute on Alcohol Abuse and Alcoholism (NIAAA), National Institute of Health (NIH), Bethesda, MD, United States; ^2^Center on Compulsive Behaviors, Intramural Research Program, National Institute of Health (NIH), Bethesda, MD, United States

**Keywords:** marijuana, cannabis, THC, CBD, CB1, AEA, 2-AG, polysomnography

## Abstract

Sleep is a vital function of the nervous system that contributes to brain and bodily homeostasis, energy levels, cognitive ability, and other key functions of a variety of organisms. Dysfunctional sleep induces neural problems and is a key part of almost all human psychiatric disorders including substance abuse disorders. The hypnotic effects of cannabis have long been known and there is increasing use of phytocannabinoids and other formulations as sleep aids. Thus, it is crucial to gain a better understanding of the neurobiological basis of cannabis drug effects on sleep, as well as the role of the endogenous cannabinoid system in sleep physiology. In this review article, we summarize the current state of knowledge concerning sleep-related endogenous cannabinoid function derived from research on humans and rodent models. We also review information on acute and chronic cannabinoid drug effects on sleep in these organisms, and molecular mechanisms that may contribute to these effects. We point out the potential benefits of acute cannabinoids for sleep improvement, but also the potential sleep-disruptive effects of withdrawal following chronic cannabinoid drug use. Prescriptions for future research in this burgeoning field are also provided.

## Introduction

The hypnogenic effects of cannabinoid drugs have long been known (Clendinning, 1843; O’Shaughnessy, 1843; Wallich, 1883; Bradbury, 1899), and there has been considerable recent interest in the use of cannabis Sativa derivatives and other cannabinoid compounds as sleep aids. This interest has coincided with renewed research on cannabis and sleep, including an emphasis on how the endogenous cannabinoids (endocannabinoids, eCBs) contribute to normal and disrupted sleep. However, there is still much to be learned about the cannabinoid/sleep relationship both in humans and experimental animals.

The eCBs are lipid metabolites that produce neuromodulatory actions mainly *via* activation of the cannabinoid type 1 (CB1) receptor (Grant and Cahn, 2005; Kano et al., 2009). The two major eCBs involved are 2-arachidonoyl-glycerol (2-AG) and arachidonoyl ethanolamide (AEA or anandamide). Activation of CB1 produces juxtacrine intracellular signals mainly through presynaptic actions on neurotransmitter release, as the receptors are expressed almost exclusively on neuronal axon terminals (Lovinger, 2008; Kano et al., 2009). The eCBs are found throughout the body and brain, as 2-AG can be produced by almost every cell type in the body, and AEA is also produced by numerous cell types. Thus, there are multiple locations within the periphery and brain at which eCBs may have actions that influence sleep.

**Figure 1:**
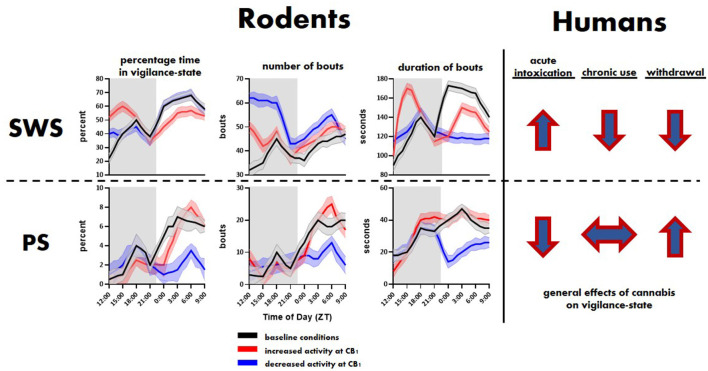
General effects of cannabinoid type 1 (CB1) activity on slow-wave sleep (SWS) and paradoxical sleep (PS) in rodents and humans. Top and bottom rows show trends related to SWS and PS, respectively. The rodent panel illustrates trends extrapolated from Pava et al. (2016). The human trends are illustrated using arrows showing the direction, i.e., overall increase or decrease, of change in SWS and PS associated with initial cannabis use, extended use and associated tolerance, and cessation of use.

The CB1 receptor is also the major target of Δ-9-tetrahydrocannabinol (THC) the major psychoactive ingredient in cannabis-derived drugs such as marijuana and hashish. This compound acts as a partial CB1 agonist. Previous studies in laboratory animals and humans indicate that THC has hypnogenic effects (Babson et al., 2017), but little is known about the cellular and circuit mechanisms underlying this action. Humans who regularly use cannabis show sleep disruption (Bolla et al., 2008, 2010; Angarita et al., 2016), particularly during withdrawal, and thus it is important to understand the mechanisms underlying effects of chronic THC and other cannabis-derived compounds such as cannabidiol (CBD) on sleep. Interactions between these compounds and eCB actions at CB1 are also likely to be important in sleep alterations (Figure 1). This review article summarizes current knowledge about cannabis/eCB effects on sleep. Future avenues in this research area are also discussed.

### A Note About Nomenclature and Sleep States

Sleep and the different phases thereof are characterized physiological using polysomnography that includes mainly electroencephalogram (EEG) or electrocorticogram (ECoG) measurements of brain electrical activity and electromyographic (EMG) measurement of muscle activity (Brown et al., 2012). This allows investigators to compare brain state and movement patterns that are now known to be characteristic of the different sleep phases. Human sleep is most often characterized as consisting of rapid-eye-movement (REM; sometimes referred to as paradoxical sleep, or PS, due to its awake state-like EEG phenotype) and slow-wave sleep (SWS, also known as non-REM or NREM) phases. Furthermore, different EEG signals have allowed investigators to separate SWS into different stages in humans, sometimes thought to correspond to the “depth” of this type of sleep (Scammell et al., 2017). In humans, REM sleep is characterized by EEG activity with prominent 3–10 Hz theta activity with diminished EMG activity (Steriade and McCarley, 1990) in some brain regions and 40–60 Hz gamma activity in the cortex that resembles awake-state activity (Horne, 2013). SWS is characterized by, as the name implies, high amplitude EEG activity in slower frequency delta ranges, a lack of EMG activity, and large bouts of higher-frequency EEG activity arising from a thalamocortical activity known as “sleep spindles” (De Gennaro and Ferrara, 2003; Iber et al., 2007). In rodents, it is not clear if there is rapid eye movement during sleep, and thus the phases with REM-like EEG and no EMG activity are often referred to as paradoxical sleep (PS; Toth and Bhargava, 2013). Furthermore, separation of the different stages of SWS sleep is not as obvious as in humans, thus this characterization is not always performed. While the term non-paradoxical sleep is sometimes used to describe NREM sleep in rodents, the term SWS is more descriptive. Thus, in this review article, we will use the terms PS and SWS to refer to the different sleep stages.

## Sleep Studies in Humans

### Cannabis and THC

It has long been suspected and was later documented, that cannabis has sleep-promoting effects (Clendinning, 1843; O’Shaughnessy, 1843; Wallich, 1883; Bradbury, 1899). Indeed, studies begun several decades ago indicated that acute exposure to cannabis or THC decreased sleep onset latency, decreased waking after sleep onset, increased slow-wave sleep and decreased REM sleep (Pivik et al., 1972; Cousens and DiMascio, 1973; Barratt et al., 1974; Feinberg et al., 1975, 1976). A 2017 survey of over 1,500 patients at a New England medical marijuana dispensary indicated that roughly two-thirds of patients decreased their use of pharmaceutical sleep medication upon medical cannabis use (Piper et al., 2017).

While indeed several studies indicate chronic exposure to THC and other CB1-activating compounds appears to produce modest sleep improvement, the quality of these current data were judged to be low in a recent Cochrane-based metanalysis (Mucke et al., 2018). Another critical review of clinical trial literature (Kuhathasan et al., 2019) stressed the need for further large scale controlled clinical trials. Still, many cannabis users cite sleep improvements as the main use motivator (Bonn-Miller et al., 2014b), and accumulating evidence suggests that cannabis use may offer therapeutic relief to individuals with poor sleep quality related to post-traumatic stress disorder (Bonn-Miller et al., 2014a, 2019; Vandrey et al., 2014; Belendiuk et al., 2015; Loflin et al., 2017) and pain (Lynch and Clark, 2003; Berman et al., 2004; Blake et al., 2006; Ware et al., 2010a; Ware et al., 2010b).

While it is evident that cannabis and THC have acute effects related to sleep, it is also clear that repeated exposure to cannabis and THC produces tolerance to drug actions, including those on sleep (Pranikoff et al., 1973; Barratt et al., 1974; Feinberg et al., 1975; Karacan et al., 1976; Freemon, 1982; Halikas et al., 1985; Gorelick et al., 2013). This can lead to the necessity for increasing dosages to obtain the sleep-promoting action. Several studies indicate that sleep is disrupted during withdrawal after chronic cannabis drug use (Karacan et al., 1976; Bolla et al., 2008, 2010; Vandrey et al., 2011). While the initial studies provided evidence for sleep disruption based on self-report by users, both older and newer studies have provided polysomnographic evidence that corresponds to subject self-report. Decreases in total sleep time, sleep efficiency, NREM, and REM sleep were observed during abstinence in heavy cannabis users (Bolla et al., 2008, 2010; Vandrey et al., 2011). Latency to sleep onset, wake after sleep, and periodic limb movements are also increased in these abstinent users.

Sleep disruption may also play a role in relapse to cannabis use following abstinence in regular users (Babson et al., 2013a, 2017). Altered sleep is observed in the majority of regular users who attempt to quit (Budney et al., 1999, 2008), and this is one of the most consistent and problematic aspects of cannabis withdrawal (Budney et al., 2001, 2003, 2008; Vandrey et al., 2008). Most of those who fail to remain abstinent list poor sleep as a major factor leading to their resumption of cannabis use (Budney et al., 2008), and those subjects slowing sleep disruption, poor sleep quality, in particular, relapse more readily (Copersino et al., 2006; Levin et al., 2010; Babson et al., 2013a,b).

It has also been suggested that cannabis may act centrally as a zeitgeber (Whitehurst et al., 2015), entraining biological rhythms to facilitate daily sleep periods, and thus may serve as a chronobiotic therapeutic compound for individuals with disrupted circadian function, such as the elderly (Hodges and Ashpole, 2019). Evidence suggests there is also chronobiological activity in the eCB system itself in humans. Endocannabinoids show circadian fluctuations in healthy humans assessed by measurement of plasma AEA and 2-AG levels (reviewed in Vaughn et al., 2010). The highest AEA plasma levels occurred upon waking and the lowest just before sleep onset. This pattern is altered by sleep disruption. However, no effect of sleep disruption was observed when AEA was measured in human cerebrospinal fluid (Koethe et al., 2009). In contrast, 2-AG levels did not show prominent circadian fluctuation. Sleep disruption was among the effects reported in the clinical trials of the CB1 antagonist/inverse agonist rimonabant (Steinberg and Cannon, 2007; Nathan et al., 2011), indicating that eCBs may contribute to sleep stability in humans. Based on these data and the findings discussed in the next section describing studies in animal models, it is clear that more research on endocannabinoids and sleep is needed.

### CBD

Most studies on the effects of cannabinoids in sleep, and in general, have focused on the effects of purified THC, or cannabis preparations with relatively high levels of THC compared to other phytocannabinoids. However, recent interest in therapeutic roles for CBD has ushered in further studies focusing on CBD, including its effects on sleep. In one study, mixed effects of combined THC and CBD have were reported, with THC generally increasing sedation and CBD having opposing, wake-enhancing effects (Nicholson et al., 2004). A long-term study of sleep quality assessed CBD actions using a common self-report instrument found a modest improvement in sleep, and more patients with improved sleep compared to poorer sleep (Shannon et al., 2019). Notably, a study using the Sativex extract formulation (containing approximately equal ~2 mg doses of THC and CBD) to examine sleep in subjects with pain-related sleep disturbances (Russo et al., 2007) reported improved sleep with no evidence of tolerance to the drug action. Thus, there may be cannabis formulations that support sustained sleep improvement. A recent controlled clinical study assessing the acute pharmacodynamic effects of 100 mg of CBD or CBD-dominant cannabis found that vapor inhalation of the latter increased subjective sleepiness. The investigators noted that CBD alone did not have significant effects on sleepiness, so possibly the effects of the CBD-dominant cannabis were due to the relatively small amounts of THC in their CBD-dominant cannabis preparation (Spindle et al., 2020). Indeed the amount of THC in their case was 3.7 mg, a similar concentration as found in many “full spectrum,” <0.3% THC, commercial CBD products (Corroon and Kight, 2018), indicating further controlled research into the physiological effects of these commercial products is necessary. It is still unclear whether acute administration of this relatively low dose of THC alone is enough to produce sleepiness, or whether it has synergistic effects with CBD and other phytocannabinoids in the strain of CBD-dominant cannabis used in this study. Currently, there is at least one larger-scale controlled clinical study to assess the effects of THC and CBD on sleep in patients with diagnosed insomnia (Suraev et al., 2020).

## Sleep Studies in Animal Models

Humans often have confounding prior experiences and pre-existing conditions, which is particularly pertinent to sleep research considering that sleep is so easily modulated by many internal and external conditions (Brower and Perron, 2010; Staner, 2010; Kalmbach et al., 2016). Animal models provide an attractive alternative to control for and overcome these concerns (Toth and Bhargava, 2013). Mice and rats are often chosen because of their wide use and availability, similarity to humans in terms of sleep neurocircuitry and neurochemistry, and, particularly in the case of mice, the potential for genetic manipulations such as transgenics, knock-ins, and knock-outs. Additionally, rodents are amenable to the recording of electrocorticogram (ECoG) signals *via* minimally invasive electrodes placed through the skull in a relatively simple surgical procedure. These ECoG signals provide strong, constant data for quantification of basic vigilance states including SWS, PS, and Wake (Mang and Franken, 2012).

However, there are some important caveats to consider when using rodents to elucidate neurobiological mechanisms of sleep phenomena. Mice and rats are polyphasic sleepers with shorter and more frequent bouts of SWS and PS, compared to humans that typically show monophasic sleep with more consolidated sleep bouts (Campbell and Tobler, 1984). Also, mice and rats spend more time sleeping (12–15 h) than humans (7–8 h) per day (see Figure 3 in Toth and Bhargava, 2013), and mice are most active nocturnally while humans are almost strictly diurnal. Despite differences in timing and architecture of sleep, the apparent similarities to human sleep have made using animal models fundamental to furthering the understanding of the neurobiology of sleep and many important advancements in treatments for sleep pathologies, including insomnia (Wisor et al., 2009), sleep apnea (Yamauchi et al., 2010), and perhaps most famously the role of orexigenic neurons in narcolepsy (Chemelli et al., 1999), can be attributed to pre-clinical research using mice and rats. With this background in mind, the following sections will review studies mainly using mice and rats to investigate cannabinergic components of sleep and sleep effects of cannabinoid drugs.

## *In Vivo* Measurements of Endocannabinoids During Sleep-Wake States

Before discussing how perturbations to the eCB system affect sleep, it is important to understand what we know about eCB activity during sleep in general. Several long-chain fatty acid molecules including those that act in eCB signaling are known to show diurnal fluctuations in neural tissue and seem to follow circadian rhythmicity (Vaughn et al., 2010). Indeed, some studies also suggest endogenous cannabinoids can act as a zeitgeber, i.e., can affect our internal circadian clock (Perron et al., 2001; Sanford et al., 2008; Acuna-Goycolea et al., 2010), indicating that the eCB system plays a role in circadian components of sleep-wake cycling. Rodent models are particularly useful for understanding how eCBs are modulated during the sleep-wake cycle, because they allow for repeated sampling *in vivo*, or collection of tissue at various timepoints, under controlled conditions. Tissue from specific brain areas that are harvested at various time-points of the laboratory lights-on and lights-off cycle (also referred to as the light/dark phase or period) can be analyzed for eCB content, typically using high-performance liquid chromatography/mass spectrometry. Cerebrospinal fluid, which can often act as a gross proxy for bulk levels of circulating molecules in the CNS, contains diurnally fluctuating levels of AEA (Murillo-Rodriguez et al., 2006a). Similarly, in the pons, a brain stem region known to be involved in sleep-wake regulation (Takata et al., 2018) and generation of REM sleep (Schwartz and Kilduff, 2015), AEA levels are low during the light-phase when rats typically spend more time sleeping, and then increase during the dark-phase, corresponding to increased arousal and wake-activities (Murillo-Rodriguez et al., 2006a). Conversely, CB1R protein expression in the pons shows diurnal variations with peak levels during lights ON periods (Martínez-Vargas et al., 2003). The interactions between diurnal fluctuations in AEA and CB1R levels in this brain stem region may contribute to transitions in or maintenance of sleep-wake states. In the hypothalamus, AEA levels are highest during the light phase (Murillo-Rodriguez et al., 2006a; Matias et al., 2008) when rats spend more time sleeping, suggesting eCB activity here might modulate diurnal variations in homeostatic related behaviors, such as feeding, associated with hypothalamic activity. In the nucleus accumbens, pre-frontal cortex, striatum, and hippocampus, structures associated with limbic and sensorimotor system functions such as learning and memory and action control, 2-AG and AEA show inverse diurnal rhythms, with the former higher during the light-phase and the latter higher during the dark-phase (Valenti et al., 2004; Matias et al., 2008), indicating the distinct role that eCBS play in sleep-wake is complex and needs further elucidation. One possible explanation for the opposing changes in AEA and 2-AG levels during light and dark periods could be that while AEA may promote sleep (Murillo-Rodriguez et al., 1998, 2001; Rueda-Orozco et al., 2010) 2-AG may promote wakefulness (Prospéro-García et al., 2016). The diurnal fluctuation of eCB levels in limbic and sensorimotor structures may reflect their complex role in awake-behavior related learning, e.g., associative learning related to action control (Lovinger and Mathur, 2012; Morena et al., 2014; Gremel et al., 2016; Mateo et al., 2017; Lupica and Hoffman, 2018), and sleep-behavior related learning, e.g., hippocampal-dependent memory consolidation (Riedel and Davies, 2005; De Oliveira Alvares et al., 2008; Yim et al., 2008; Busquets-Garcia et al., 2016).

## Effects of Endocannabinoid System Perturbations on Sleep in Rodents

In addition to facilitating *in vivo* and *ex vivo* measurements of diurnal eCB activity and how these measurements correlate to sleep-wake states, rodent models allow for more causal experimentation. There are several manipulations, both externally (pharmacologic), and internally (genetic) that can be used in rodent models to affect eCB tone and eCB system function and examine the consequences for sleep.

### Pharmacologic Approaches

Typical pharmacologic manipulations include treatment with drugs that either enhance or diminish endocannabinoid tone. Enhancing eCB tone can be accomplished by administering 2-AG and AEA themselves, or by administering agents that increase the amount of these eCBs in the synapse *via* increasing their synthesis and release, or by inhibiting the proteins that contribute to their reuptake and breakdown. In contrast, decreasing eCB activity in the brain can result from compounds that block eCBs from binding to CB1, or inhibiting their synthesis and release.

### CB1 Agonists and Antagonists

Systemic administration of AEA is known to promote sleep (Mechoulam et al., 1997; Murillo-Rodriguez et al., 1998, 2001, 2003), an effect which is partially due to its action in the pedunculopontine tegmental nucleus (PPTg), a hindbrain region implicated in sleep and arousal mechanisms (Murillo-Rodriguez et al., 2001). The effect of both systemic and intra-PPTg AEA administration on sleep is blocked by pretreatment with the CB1 receptor antagonist/inverse agonist SR141716A (Murillo-Rodriguez et al., 2001). Other sleep-promoting effects of systemic AEA administration could also be driven by increases in basal forebrain adenosine (Murillo-Rodriguez et al., 2003), one of the hallmarks of normal wake-sleep transitions (Blanco-Centurion et al., 2006). Direct injection of 2-AG into the lateral hypothalamus, a brain region that contains both awake-promoting orexin neurons and sleep-promoting melanin-concentrating hormone (MCH) neurons (Yamashita and Yamanaka, 2017), has been shown to increase REM sleep, likely by increasing activity in MCH-producing neurons (Perez-Morales et al., 2013). This treatment also mitigates sleep disruption due to early-life stress in rats (Perez-Morales et al., 2014). The hippocampus is also known to be involved in sleep and its role in memory consolidation (Marshall and Born, 2007). Intrahippocampal administration of AEA during the dark phase, but not the light phase, has been shown to increase REM sleep; an effect that is blocked by the CB1 antagonist/inverse agonist AM251 (Rueda-Orozco et al., 2010). Systemic administration of the antagonist/inverse agonist SR141716A itself has also been shown to increase time spent in wakefulness, and decrease SWS and REM in a dose-dependent manner, in addition to reducing the spectral power of ECoG SWS signals (Santucci et al., 1996).

### Enzyme Modulation

In addition to using systemic or intracranial administration of direct CB1 antagonists and antagonists, several studies have targeted the proteins and enzymes that modulate eCB tone and investigated their effects on sleep and sleep circuitry. Intracerebroventricular (i.c.v) or intraperitoneal (i.p.) injection of the selective fatty acid amide hydrolase (FAAH) inhibitor, URB597, which leads to an accumulation of AEA in the CNS, counterintuitively produced a decrease in SWS and increase in wakefulness. Also, this treatment increased c-Fos immunoreactivity in wake-promoting regions in the hypothalamus and dorsal raphe nucleus, and increased extracellular levels of dopamine in the nucleus accumbens (NAc; Murillo-Rodriguez et al., 2007, 2016). These latter findings may be related to fluctuations in endogenous NAc dopamine (DA) levels during innate sleep-wake cycling in rodents (Lena et al., 2005). Additional studies show that direct micro-infusions of URB597 into lateral hypothalamus or dorsal raphe alone is sufficient to recapitulate wake-promoting and DA-increasing effects seen with i.c.v. administration (Murillo-Rodriguez et al., 2011b). The wake-promoting effects of FAAH inhibition using URB579 in these studies contradict the idea that AEA promotes sleep, and similar studies show that systemic injection of the same URB compound failed to produce a substantial effect on sleep. Rather the more potent FAAH inhibitor AM3506 significantly increases NREM sleep (Pava et al., 2016). The inconsistency in the effects of FAAH antagonism on sleep could be due to differences in routes of administration, dose, and timing of treatments with respect to the light-dark phase. Additionally, factors such as receptor desensitization following prolonged AEA exposure (Zhuang et al., 1998; Garzon et al., 2009) may also be involved in drug effects.

In addition to inhibiting FAAH, it is possible to increase the AEA tone by blocking its reuptake into cells and thereby promoting its intercellular effects. The selective AEA transporter inhibitor, VDM11, when administered i.c.v. or i.p. at the beginning of the light-phase, potentiated sleep, and decreased wakefulness (Murillo-Rodriguez et al., 2008a,b, 2016), an effect recapitulated by infusing VDM11 or a different AEA uptake inhibitor, OMDM-2, directly into the paraventricular thalamic nucleus. These effects were correlated with decreases in extracellular DA levels in the NAc (Murillo-Rodriguez et al., 2013), and are consistent with a hypnogenic effect of AEA increase.

Our group has performed the only study on the effect on sleep of increasing 2-AG tone *via* inhibiting its degradation enzyme monoacylglycerol lipase (MAGL) using JZL184 (Pava et al., 2016). Systemic administration of this compound profoundly augmented SWS sleep while only slightly reducing PS when administered just before dark-phase, while having a much more modest effect on SWS and stronger effect on PS when administered before light-phase. These data support a role for 2-AG in promoting sleep, as might be expected considering previously discussed studies showing 2-AG increases during the light-phase when rodents spend more time sleeping. However, increasing endogenous 2-AG tone using JZL184 seems to produce effects on REM sleep opposite to those observed following direct infusion of 2-AG into the lateral hypothalamus as described in the previous section, and further research is needed to understand the basis of this inconsistency. It is most likely the case that 2-AG has sleep-promoting effects in some brain regions, but wake-promoting effects in other areas, due to the various synapses it modulates.

Overall, there seems to be a clear consensus in these studies that modulating eCB actions by administering direct agonists or antagonists of the CB1 receptor, or by modulating enzymes involved in their endogenous tone, has profound effects on the time spent in various vigilance states. However, there appear to be some inconsistencies in the literature as to the direction, either increase or decrease, of these effects. These inconsistencies are likely due to the differences in routes of administration and doses of the compounds given. Additionally, many of these studies only examine the effects of these treatments on sleep for shorter periods, on the order of 4–8 h, and report data compiled into particularly large time bins, making it difficult to interpret the role of eCBs on the vigilance-state architecture that contribute to overall times spent in each state. Below we summarize studies from our group that address these issues.

## eCBs Mediate Sleep Stability

In a set of experiments aimed to address the poor consensus as to the effects of eCB signaling in sleep and underlying vigilance-state architecture, our group performed experiments that totaled over 11,000 h of ECoG/EMG recordings in mice after different treatments targeting the eCB system (Pava et al., 2016). In these experiments, pharmacologic agents targeting the CB1 receptor itself or the enzymes MAGL/FAAH were administered before both the dark-phase and light-phase. Data was recorded for at least 24 h and within-subject comparisons of baseline, vehicle, and treatment recordings were conducted. In addition to fine-scale analysis of vigilance state architecture including time spent in the state, and the number and duration of bouts, the spectral power densities of each state were also reported. The main findings were that the eCB system plays a role in modulating the stability of vigilance states, rather than driving changes in sleep *via* mechanisms that are mediated by sleep-homeostasis. The finer-scale analysis of vigilance-state architecture showed that direct activation of CB1 augmented SWS by stabilizing and increasing the duration of individual bouts, while blockade of CB1 using AM281 fragmented SWS. Conversely, REM sleep was suppressed upon activation of CB1 either with direct agonists or MAGL/FAAH inhibition. Importantly, it was shown that increasing eCB signaling had biphasic effects, where manipulations that augmented sleep during the dark-phase had secondary effects of decreasing sleep during the light-phase, all while not altering overall sleep time. This secondary effect is a normal homeostatic response to increased sleep during the dark-phase and indicates that homeostatic machinery remains intact despite perturbations to eCB the eCB system. These results, taken with an additional experiment showing that CB1 blockade had a negligible effect on sleep time and amount of sleep after a period of total sleep deprivation, indicate that eCB signaling is involved in regulating sleep stability rather than mediating sleep homeostatic drive.

### Genetic Manipulations

In addition to pharmacologic treatments, a handful of studies have employed transgenic techniques to genetically modify mice to over or under-express, or knock-out, genes that play a role in eCB synthesis, degradation, and signaling to examine the effects of these eCB system components on sleep architecture. Mice lacking the gene coding for CB1 protein have reduced and fragmented SWS, a mild reduction in PS sleep, and spend more time awake. They also show alterations in the SWS power spectrum (Pava et al., 2014; Silvani et al., 2014), which is thought to be due to increased cortical excitability (Pava et al., 2014). However, these studies are subject to confounds due to developmental abnormalities in the CB1 knockout mice (Zimmer et al., 1999; Wu et al., 2010). As expected, conversely to CB1 knockout mice, mice lacking the gene for FAAH show augmented SWS that stems from increased SWS bout duration, while maintaining normal sleep homeostasis in response to sleep deprivation (Huitron-Resendiz et al., 2004). Further research is needed to better understand how genetic abnormalities in the eCB system may affect sleep, and these studies should use the power of transgenic/knockout mouse models, e.g., Cre/Lox, to perform this research in a more targeted manner.

## Phytocannabinoids and Sleep in Rodents

**Figure 2:**
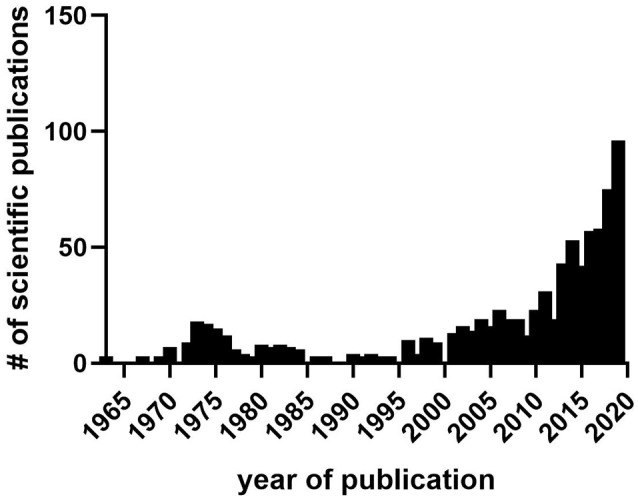
The number of scientific publications in PubMed containing *sleep* and either *cannabis*, Δ-9-tetrahydrocannabinol *(THC)*, cannabidiol *CBD*, or *cannabinoid* from 1963 to 2019. Data collected from PubMed in March 2020. Notice lull in research publications during the 1980s–1990s between prior interest in the 1970s stemming from initial findings on hypnogenic properties of cannabis and its distillates, and later interest after the discovery of the endocannabinoid system and identification of sleep effects during cannabis use, abuse, and withdrawal.

As mentioned in the “Introduction” section the hypnogenic characteristics of cannabinoids derived from plants, i.e., phytocannabinoids, particularly *Cannabis sativa*, have been known and exploited for centuries by humans (Clendinning, 1843; O’Shaughnessy, 1843; Wallich, 1883; Bradbury, 1899). As described above in the section on human studies, there is accumulating evidence that phytocannabinoids can produce profound effects on both subjective, e.g., individual reports of sleep quality, and objective, e.g., alterations in polysomnographic recordings, measurements. However, the neurobiological mediators of these effects remain unknown and are difficult to study in humans. However, pre-clinical research has offered insight into the central actions of the main phytocannabinoid derivatives in cannabis, THC, and CBD, in altering sleep-wake states and physiology. Studies using EEG recordings in mice, rats, rabbits, cats, and monkeys began in the late 1960s with reports that, in general, cannabis extracts containing a myriad of phytocannabinoid compounds produced an augmentation of SWS and a reduction of PS that showed some evidence of tolerance after chronic treatment regimens (Masur and Khazan, 1970; Barratt and Adams, 1973, 1975; Wallach and Gershon, 1973; Willinsky et al., 1973). Among these early reports, several studies showed synergistic effects of a cannabis extract, THC, and CBD with sleep caused by anesthetics and other potent hypnogenic compounds (Bose et al., 1963; Garriott et al., 1967; Kubena and Barry, 1970; Paton and Pertwee, 1972; Rating et al., 1972; Chesher et al., 1974; Friedman and Gershon, 1974; Siemens et al., 1974; Oishi et al., 1988). The sleep-prolonging effects of phytocannabinoids in concert with hypnogenics provided early evidence that the still undiscovered eCB system would play a role in the neurobiology of sleep. Subsequently, both preclinical and clinical interest in the interaction between phytocannabinoids and sleep fell during the 1980s and 1990s, possibly due to difficulties in obtaining these compounds due to their federal legal classification. However, recent years have seen a resurgence in this research area (Figure 2), potentially due to clinical indications of sleep disturbances being a major factor in cannabis use disorder and cannabis withdrawal (Haney et al., 1999a,b; Budney et al., 2003, 2004; Vandrey et al., 2011; Gates et al., 2016). In the past two-decades, pre-clinical research has seemingly struggled to expound upon the many clinical reports on phytocannabinoid use and sleep, as it is often still difficult for research laboratories to obtain these agents, however, the current climate towards legalization will hopefully allow more research to be conducted. The majority of recent studies have focused on understanding the individual contributions of either THC or CBD. Indeed, several recent studies have used cannabis extracts (Mondino et al., 2019), purified THC (Kimura et al., 2019), or CBD (Murillo-Rodriguez et al., 2006b, 2008b, 2011a, 2014) to study how acute administration of these compounds affects sleep and sleep-pathologies including sleep apnea (Carley et al., 2002; Calik et al., 2014) and depression increased PS (Hsiao et al., 2012). This increased productivity in animal model studies of phytocannabinoid actions is likely to accelerate and will provide much-needed neurobiological information about the mechanisms involved in the actions of these substances. Also, this work will provide a sound scientific basis for decisions about the use of phytocannabinoids as sleep aids.

## Molecular Mechanisms Potentially Underlying Cannabinoid Effects on Sleep

It is not yet clear which eCB has a more prominent role in the hypnotic effects of eCBs, and indeed both 2-AG and AEA may be involved. As mentioned in previous sections, acutely elevated levels of either eCB by inhibiting the enzymes responsible for catabolism increased NREM sleep and affects sleep stability in mice (Pava et al., 2016). Given that effects on sleep differ with acute vs. chronic use of cannabis and cannabinoid pharmaceuticals, it would be interesting to study how chronic disruption of FAAH or MAGL activity affects sleep. To our knowledge, no such studies have been conducted.

Similarly, further studies are needed to determine if altering eCB production, rather than catabolism, reduces or disrupts sleep, though certainly blocking their endogenous action on CB1 has sleep affects. This will be more easily accomplished for 2-AG, as abundant tools are available for inhibition or genetic disruption of DAG lipase activity. Unfortunately, there is still uncertainty about the major AEA synthesis pathway (Lu and Mackie, 2016), and thus it will be difficult to assess the effects of disrupting the production of this eCB. Another significant question that should drive future research is the brain regional and synaptic location of eCB signaling important for sleep. The observation that peripheral manipulations of eCB signaling have an overall hypnogenic effect suggests that there may not be opposing mechanisms related to sleep. Indeed, there may be several brain regions in which eCBs act to promote sleep. We will return to the discussion of possible brain regions involved in eCB hypnotic effects later in this section of the review.

**Figure 3:**
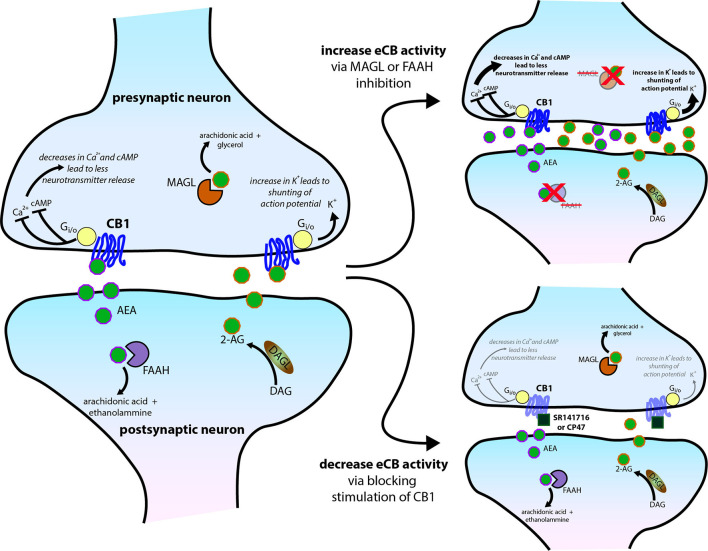
Molecular mechanisms of central cannabinoid action. Schematic diagram of the endocannabinoid (eCB) system perturbations that have been used in experiments and have altered sleep. eCBs, either anandamide/N-arachidonoylethanolamine (AEA) or 2-arachidonoylglycerol (2-AG), generated and then released from the postsynaptic neuron typically act on presynaptic CB1 receptors to reduce presynaptic neurotransmitter release *via* several intracellular signaling cascades. AEA and 2-AG are then typically catabolized and removed from the system by fatty acid amide hydrolase (FAAH) and monoacylglycerol lipase (MAGL), respectively. See Figure 1 for trends in sleep that can occur from the two scenarios further illustrated on the right side of the schematic.

The eCB roles in sleep involve the CB1 receptor (Figure 3). As Pava et al. (2016) showed, antagonism of this receptor eliminates the sleep-promoting effects of acute eCB elevation and CB1 agonists. Also, the application of CB1 inverse agonists reduced NREM sleep, indicating a role for CB1-mediated eCB signaling in sleep promotion even in the absence of pharmacological enhancement of 2-AG or AEA signaling. It is likely that THC also produces acute sleep-promoting and chronic sleep-disrupting effects through its known actions at CB1. The intracellular mechanisms proximal to CB1 activation are well known, and likely to be the first steps in sleep promotion. The liberation of the Gα_i/o_ G-protein subunit inhibits Adenylyl cyclase (AC), the enzyme that catalyzes the production of cycle adenosine-monophosphate (cAMP; Filipek et al., 2012), whose signaling is a highly conserved component of sleep regulation (Zimmerman et al., 2008) and known to be altered upon sleep-deprivation (Vecsey et al., 2009; Havekes et al., 2012). Inhibition of cAMP production is thought to decrease neurotransmitter release by reducing the activity of protein kinase A (PKA) and altered phosphorylation of vesicle-associated proteins such as Rim1 (Lonart et al., 2003; Chevaleyre et al., 2007). This intracellular signaling pathway has also been linked to protein synthesis in presynaptic terminals that is involved in long-term synaptic depression (Younts et al., 2016). Receptor-mediated liberation of the beta/gamma subunits allows this complex to bind directly to voltage-gated calcium channels of the N (containing the CaV2.2 alpha subunit) and P/Q (containing the CaV2.1 alpha subunit) types to reduce the calcium entry needed for excitation/secretion coupling. A similar mechanism is involved in activation of the G-protein-activated inwardly-rectifying potassium (GIRK) ion channel (Betke et al., 2012). This channel activation mechanism may also shunt action potentials entering the axon terminal, thus reducing calcium influx indirectly. Also, beta/gamma subunits interact with proteins involved in vesicle fusion, yielding a direct inhibition of the fusion process (Yim et al., 2018). Thus, the predominantly presynaptic CB1 receptor can reduce neurotransmitter release *via* different mechanisms, with the beta/gamma subunit-mediated processes yielding fast and transient reductions, and the alpha subunit/AC/PKA mechanisms likely involved in longer-term reductions that outlast receptor occupancy (Monday et al., 2018). Indeed, reduction in neuronal activity, either by blocking excitatory action or increasing inhibitory tone, is a hallmark of many classical hypnogenic compounds including benzodiazepines and alcohol, and more recent non-benzodiazepine-type hypnotics like zolpidem (Smith, 1977; Doble, 1999; Sanger, 2004; Hammer et al., 2015).

The CB1 receptor has also been reported to alter intracellular signaling in glia (Navarrete and Araque, 2008, 2010; Stella, 2010; Han et al., 2012) and some postsynaptic neuronal elements (Ong and Mackie, 1999; Bacci et al., 2004; see Cachope, 2012 and Castillo et al., 2012 for further information on this eCB signaling modalities). These mechanisms should also be considered in exploring the range of receptor actions that could be involved in affecting sleep. For example, a recent study found that neurons in the suprachiasmatic nucleus, a hypothalamic region critical for controlling the circadian rhythm, release eCBs that activate intracellular astrocyte signaling which subsequently influences circadian clock timing (Hablitz et al., 2020).

Receptor signaling independent of G-proteins has been characterized for several GPCRs, including CB1 (Rajagopal et al., 2010), although this concept is still somewhat controversial (Grundmann et al., 2018). The best-characterized signaling process of this type involves receptor internalization followed by phosphorylation of the receptor that is catalyzed by GPCR receptor kinases (GRKs; Rajagopal et al., 2010). This phosphorylation in turn promotes the binding of beta-arrestin to the receptor to initiate receptor internalization and entry into recycling/degradation pathways (Priestley et al., 2017). This molecular mechanism can also promote intracellular signaling including phosphorylation of the extracellular signaling-related kinase (ERK, aka MAPK) and other phosphoproteins that promote long-term intracellular changes and can affect gene-regulation mechanisms (Rajagopal et al., 2010). It should be noted that these effects are generally engaged by prolonged receptor occupancy. Thus, signaling through such pathways may be a good candidate for sleep disruption following chronic exposure to THC or chronically-elevated eCB levels.

Adaptations in synaptic transmission produced by chronic THC exposure provide information about mechanisms that could contribute to sleep disruption. There is considerable physiological evidence that CB1 receptor inhibition of neurotransmitter release is reduced or lost following chronic THC exposure (Hoffman et al., 2003, 2007; Mato et al., 2005; Nazzaro et al., 2012). This functional tolerance has been observed at both glutamatergic and GABAergic synapses, and in brain regions including the dorsal striatum, hippocampus, and NAc. The molecular mechanisms taking place within axon terminals that mediate the loss of synaptic modulation are not fully understood, mainly due to the difficulty of measuring intracellular signaling in these tiny subcellular components. However, several candidate mechanisms for tolerance have been suggested based on the measurement of signaling molecules and manipulation of signaling pathways both *in vivo* and in brain slice and cell culture preparations.

Indeed, reduced CB1 radioligand binding has been observed in the cerebellum, cerebral cortex, hippocampus, globus pallidus, spinal cord, striatum, and other brain regions following chronic agonist exposure (Romero et al., 1997; Breivogel et al., 1999; Rubino et al., 2000, 2004; Tonini et al., 2006; Tappe-Theodor et al., 2007; Martini et al., 2010). The most convincing evidence for agonist-induced decreases in CB1 at specific presynaptic terminals comes from the work of Mikasova et al. (2008) who examined receptor mobility in cortical neurons using single-molecule tracking, and Dudok et al. (2015) who used STORM super-resolution microscopy to image and quantify CB1 on GABAergic terminals in the CA1 region of the hippocampus. In the latter study chronic THC exposure led to a clear decrease in the receptors on these terminals. Several mechanisms for CB1 downregulation have been explored. The aforementioned role for beta-arrestin in signaling by internalized receptors may also play a role in altering recycling and degradation. Exposure to THC increases the expression of several GRK and beta-arrestin subtypes (Rubino et al., 2006). Altering beta-arrestin expression and signaling has also been shown to alter molecular and behavioral indices of cannabinoid tolerance (Nguyen et al., 2012), but mixed effects have been observed in different studies (Nguyen et al., 2012; Breivogel et al., 2013; Breivogel and Vaghela, 2015).

Receptor-associated proteins have also been implicated in CB1 downregulation and tolerance. The GASP1 protein is implicated in agonist-induced downregulation of several GPCRs including CB1 (Martini et al., 2010). Mice that lack GASP1 did not show agonist-induced downregulation of CB1, and the protein has been implicated in agonist-induced receptor internalization and behavioral tolerance (Tappe-Theodor et al., 2007; Martini et al., 2010). The Src homology 3-domain growth factor receptor-bound 2-like (endophilin) interacting protein 1 (SGIP1) appears to interact with CB1 at the C-terminus. This binding inhibits clathrin-mediated CB1 endocytosis and enhances receptor interaction with beta-arrestin2 (Hajkova et al., 2016). The CRIP protein has also been postulated to interact with CB1 and reduce receptor internalization (Smith et al., 2015).

Among the intracellular signaling pathways implicated in tolerance to CB1 activation are those involving cAMP/PKA and ERK. As previously mentioned, acute CB1 activation generally inhibits AC leading to decreased PKA activation. This effect may be lost or diminished following chronic agonist exposure, and indeed increased cAMP levels have been observed following such treatment (Rubino et al., 2000; Tzavara et al., 2000). In the cerebellum, the timing of the increase in cAMP levels parallels that of the onset of antagonist-precipitated withdrawal signs (Tzavara et al., 2000). These investigators also showed that inhibiting PKA in the cerebellum reduced the severity of withdrawal. Chronic exposure to low-dose THC resulted in the upregulation of the cAMP/PKA-activated phospho-CREB transcription factor in the hippocampus (Fishbein et al., 2012). Thus, activation of cAMP/PKA signaling during chronic THC exposure likely alters the expression of genes that can have wide-ranging effects on synaptic transmission and neuronal function, potentially affecting central control of sleep.

There is also considerable evidence that the ERK pathway contributes to CB1 tolerance following *in vivo* agonist exposure. As mentioned above, receptor activation, including THC, increases ERK phosphorylation. Activation of ERK may involve receptor internalization and beta-arrestin mechanisms, as discussed previously (Rajagopal et al., 2010), but inhibition of AC can also result in ERK phosphorylation/activation (Davis et al., 2003). This activation continues during chronic THC exposure in brain regions including the cerebellum, hippocampus, and prefrontal cortex (Rubino et al., 2004; Tonini et al., 2006). In Ras-GRF1-KO mice, receptor activation of ERK is lost, and these mice show deficits in tolerance to the sedative and analgesic effects of THC (Rubino et al., 2004). Several other signaling pathways are also altered by chronic THC exposure, including c-FOS and delta-FosB expression and production of brain-derived neurotrophic factor (Fishbein et al., 2012). Chronic THC exposure has also been shown to alter AMPA and NMDA receptor expression and function in postsynaptic elements of neurons in the hippocampus, and these changes were accompanied by reduced long-term potentiation and impaired fear memory and spatial learning and memory (Chen et al., 2013). These findings indicate that neuroadaptations to prolonged THC exposure are not limited to the presynaptic site of CB1 actions but may have more general effects on synaptic function and plasticity. Upregulation of COX2, an enzyme involved in eCB degradation has also been observed following chronic THC exposure (Chen et al., 2013). Thus, prolonged receptor activation may impact subsequent eCB signaling.

Unfortunately, it is still unclear if any of the chronic THC-induced signaling changes take place in presynaptic terminals, as they have not been measured within terminals themselves. It is presumed that the decrease in CB1 radioligand binding and other evidence of decreased receptors following chronic THC reflects decreased expression on the membranes in axon terminals as the bulk of receptors reside on terminals. However, this is not fully established, and it is not clear which terminals exhibit decreased receptor numbers, except for the clear evidence from the Mikasova et al. (2008) and Dudok et al. (2015) studies discussed previously. Additional use of powerful subcellular imaging techniques will likely be needed to determine how CB1 and associated signaling molecules change in following THC exposure.

It should be noted that some studies did not detect changes in tissue CB1 receptor levels in following chronic THC exposure despite evidence of behavioral or physiological tolerance (Abood et al., 1993; Mato et al., 2005). In other studies very small changes in binding were observed in a brain region (the striatum in this case) in which functional tolerance was clear (Breivogel et al., 1999). Another phenomenon that cannot be explained simply by a decrease in cell surface CB1 receptors is cross-tolerance with the mu-opioid receptor. Chronic THC exposure results in loss of synaptic depression induced by a mu opiate receptor agonist (Hoffman et al., 2003). This cross-tolerance can also be observed following *in vivo* treatment with opioid drugs that reduce CB1 agonist-mediated synaptic depression in the dorsal striatum (Atwood et al., 2014). Thus, more general changes in presynaptic G_i/o_ signaling might be induced by chronic THC, perhaps involving the intracellular signaling mechanisms mentioned above.

While, we presume that the sleep-altering molecular mechanisms engaged during acute and chronic THC exposure take place in circuits controlling sleep and wake, this remains to be examined in many of the brain regions with important roles in these processes and should be an area of considerable further research.

## Conclusions

It is becoming increasingly evident that endocannabinoids play a prominent role in sleep and sleep neurophysiology, and cannabinoid drugs alter these processes. There are clear overlaps between the brain eCB system and sleep-wake circuitry, and cannabinergic manipulations are capable of altering sleep on a large scale in terms of time spent in specific vigilance states, and fine-scale in terms of sleep architecture and spectral power of specific sleep-related brain rhythms. Given that a significant portion of the population suffers from poor sleep quality or sleep-related disorders (DSM-V; American Psychiatric Association, 2013), with an estimated 50–70 million individuals in the United States alone as of 2006 (Colten and Altevogt, 2006), understanding how eCBs are functioning under normal and pathological conditions can offer insight to these illnesses and potential treatments. Additionally, the current societal climate moving towards legalization of cannabis use for recreational purposes and the already prominent cannabis use for medical reasons, including treating sleep issues, make it increasingly pertinent to understand how phytocannabinoids interact with the eCB system to alter sleep and the therapeutic nature of these effects. We feel that continued clinical research and further back-translational efforts to model cannabinoid mediated sleep alterations, and the molecular mechanisms of cannabinoid actions in sleep-related neurons and circuits, will be fruitful in understanding how cannabinoids influence sleep, in addition to furthering our understanding the basic biology of sleep-wake states.

## Author Contributions

AK and DL contributed equally to writing this manuscript.

## Conflict of Interest

The authors declare that the research was conducted in the absence of any commercial or financial relationships that could be construed as a potential conflict of interest.

## References

[B1] Abood M. E. Sauss C. Fan F. Tilton C. L. Martin B. R. (1993). Development of behavioral tolerance to Δ9-THC without alteration of cannabinoid receptor binding or mRNA levels in whole brain. Pharmacol. Biochem. Behav. 46, 575–579. 10.1016/0091-3057(93)90546-6 8278434

[B2] Acuna-Goycolea C. Obrietan K. van den Pol A. N. (2010). Cannabinoids excite circadian clock neurons. J. Neurosci. 30, 10061–10066. 10.1523/jneurosci.5838-09.2010 20668190 PMC2927117

[B3] Angarita G. A. Emadi N. Hodges S. Morgan P. T. (2016). Sleep abnormalities associated with alcohol, cannabis, cocaine and opiate use: a comprehensive review. Addict. Sci. Clin. Pract. 11:9. 10.1186/s13722-016-0056-7 27117064 PMC4845302

[B4] American Psychiatric Association (2013). Diagnostic and Statistical Manual of Mental Disorders, 5th Edition. Arlington, VA: American Psychiatric Association.

[B5] Atwood B. K. Kupferschmidt D. A. Lovinger D. M. (2014). Opioids induce dissociable forms of long-term depression of excitatory inputs to the dorsal striatum. Nat. Neurosci. 17, 540–548. 10.1038/nn.3652 24561996 PMC4163916

[B7] Babson K. A. Boden M. T. Bonn-Miller M. O. (2013a). The impact of perceived sleep quality and sleep efficiency/duration on cannabis use during a self-guided quit attempt. Addict. Behav. 38, 2707–2713. 10.1016/j.addbeh.2013.06.012 23906725

[B8] Babson K. A. Boden M. T. Harris A. H. Stickle T. R. Bonn-Miller M. O. (2013b). Poor sleep quality as a risk factor for lapse following a cannabis quit attempt. J. Subst. Abuse Treat. 44, 438–443. 10.1016/j.jsat.2012.08.224 23098380

[B6] Babson K. A. Sottile J. Morabito D. (2017). Cannabis, cannabinoids and sleep: a review of the literature. Curr. Psychiatry Rep. 19:23. 10.1007/s11920-017-0775-9 28349316

[B9] Bacci A. Huguenard J. R. Prince D. A. (2004). Long-lasting self-inhibition of neocortical interneurons mediated by endocannabinoids. Nature 431, 312–316. 10.1038/nature02913 15372034

[B10] Barratt E. S. Adams P. M. (1973). Chronic marijuana usage and sleep-wakefulness cycles in cats. Biol. Psychiatry 6, 207–214. 4354505

[B11] Barratt E. S. Adams P. M. (1975). Effect of chronic marijuana administration of stages of primate sleep-wakefulness. Biol. Psychiatry 10, 315–322. 166711

[B12] Barratt E. S. Beaver W. White R. (1974). The effects of marijuana on human sleep patterns. Biol. Psychiatry 8, 47–54. 4361028

[B13] Belendiuk K. A. Babson K. A. Vandrey R. Bonn-Miller M. O. (2015). Cannabis species and cannabinoid concentration preference among sleep-disturbed medicinal cannabis users. Addict. Behav. 50, 178–181. 10.1016/j.addbeh.2015.06.032 26151582

[B14] Berman J. S. Symonds C. Birch R. (2004). Efficacy of two cannabis based medicinal extracts for relief of central neuropathic pain from brachial plexus avulsion: results of a randomised controlled trial. Pain 112, 299–306. 10.1016/j.pain.2004.09.013 15561385

[B15] Betke K. M. Wells C. A. Hamm H. E. (2012). GPCR mediated regulation of synaptic transmission. Prog. Neurobiol. 96, 304–321. 10.1016/j.pneurobio.2012.01.009 22307060 PMC3319362

[B16] Blake D. R. Robson P. Ho M. Jubb R. W. McCabe C. S. (2006). Preliminary assessment of the efficacy, tolerability and safety of a cannabis-based medicine (Sativex) in the treatment of pain caused by rheumatoid arthritis. Rheumatology 45, 50–52. 10.1093/rheumatology/kei183 16282192

[B17] Blanco-Centurion C. Xu M. Murillo-Rodriguez E. Gerashchenko D. Shiromani A. M. Salin-Pascual R. J.. (2006). Adenosine and sleep homeostasis in the Basal forebrain. J. Neurosci. 26, 8092–8100. 10.1093/sleep/29.11.1387 16885223 PMC6673779

[B18] Bolla K. I. Lesage S. R. Gamaldo C. E. Neubauer D. N. Funderburk F. R. Cadet J. L.. (2008). Sleep disturbance in heavy marijuana users. Sleep 31, 901–908. 10.1093/sleep/31.6.901 18548836 PMC2442418

[B19] Bolla K. I. Lesage S. R. Gamaldo C. E. Neubauer D. N. Wang N. Y. Funderburk F. R.. (2010). Polysomnogram changes in marijuana users who report sleep disturbances during prior abstinence. Sleep Med. 11, 882–889. 10.1016/j.sleep.2010.02.013 20685163 PMC2938870

[B21] Bonn-Miller M. O. Babson K. A. Vandrey R. (2014a). Using cannabis to help you sleep: heightened frequency of medical cannabis use among those with PTSD. Drug Alcohol Depend. 136, 162–165. 10.1016/j.drugalcdep.2013.12.008 24412475 PMC3929256

[B22] Bonn-Miller M. O. Boden M. T. Bucossi M. M. Babson K. A. (2014b). Self-reported cannabis use characteristics, patterns and helpfulness among medical cannabis users. Am. J. Drug Alcohol Abuse 40, 23–30. 10.3109/00952990.2013.821477 24205805

[B20] Bonn-Miller M. O. Pollack C. V. Jr. Casarett D. Dart R. ElSohly M. Good L.. (2019). Priority considerations for medicinal cannabis-related research. Cannabis Cannabinoid Res. 4, 139–157. 10.1089/can.2019.0045 31579832 PMC6757233

[B23] Bose B. C. Saifi A. Q. Bhagwat A. W. (1963). Effect of cannabis indica on hexobarbital sleeping time and tissue respiration of rat brain. Arch. Int. Pharmacodyn. Ther. 141, 520–524. 14014152

[B24] Bradbury J. B. (1899). The croonian lectures on some points connected with sleep, sleeplessness and hypnotics: delivered before the royal college of physicians of london. Br. Med. J. 2, 134–138. 10.1136/bmj.1.2008.1528 20758585 PMC2411681

[B26] Breivogel C. S. Childers S. R. Deadwyler S. A. Hampson R. E. Vogt L. J. Sim-Selley L. J. (1999). Chronic Δ9-tetrahydrocannabinol treatment produces a time-dependent loss of cannabinoid receptors and cannabinoid receptor-activated G proteins in rat brain. J. Neurochem. 73, 2447–2459. 10.1046/j.1471-4159.1999.0732447.x 10582605

[B27] Breivogel C. S. Puri V. Lambert J. M. Hill D. K. Huffman J. W. Razdan R. K. (2013). The influence of beta-arrestin2 on cannabinoid CB1 receptor coupling to G-proteins and subcellular localization and relative levels of beta-arrestin1 and 2 in mouse brain. J. Recept. Signal Transduct. Res. 33, 367–379. 10.3109/10799893.2013.838787 24094141

[B25] Breivogel C. S. Vaghela M. S. (2015). The effects of beta-arrestin1 deletion on acute cannabinoid activity, brain cannabinoid receptors and tolerance to cannabinoids in mice. J. Recept. Signal Transduct. Res. 35, 98–106. 10.3109/10799893.2014.1003659 25779032

[B28] Brower K. J. Perron B. E. (2010). Sleep disturbance as a universal risk factor for relapse in addictions to psychoactive substances. Med. Hypotheses 74, 928–933. 10.1016/j.mehy.2009.10.020 19910125 PMC2850945

[B29] Brown R. E. Basheer R. McKenna J. T. Strecker R. E. McCarley R. W. (2012). Control of sleep and wakefulness. Physiol. Rev. 92, 1087–1187. 10.1152/physrev.00032.2011 22811426 PMC3621793

[B31] Budney A. J. Hughes J. R. Moore B. A. Novy P. L. (2001). Marijuana abstinence effects in marijuana smokers maintained in their home environment. Arch. Gen. Psychiatry 58, 917–924. 10.1001/archpsyc.58.10.917 11576029

[B32] Budney A. J. Hughes J. R. Moore B. A. Vandrey R. (2004). Review of the validity and significance of cannabis withdrawal syndrome. Am. J. Psychiatry 161, 1967–1977. 10.1176/appi.ajp.161.11.1967 15514394

[B30] Budney A. J. Moore B. A. Vandrey R. G. Hughes J. R. (2003). The time course and significance of cannabis withdrawal. J. Abnorm. Psychol. 112, 393–402. 10.1037/0021-843x.112.3.393 12943018

[B33] Budney A. J. Novy P. L. Hughes J. R. (1999). Marijuana withdrawal among adults seeking treatment for marijuana dependence. Addiction 94, 1311–1322. 10.1046/j.1360-0443.1999.94913114.x 10615717

[B34] Budney A. J. Vandrey R. G. Hughes J. R. Thostenson J. D. Bursac Z. (2008). Comparison of cannabis and tobacco withdrawal: severity and contribution to relapse. J. Subst. Abuse Treat. 35, 362–368. 10.1016/j.jsat.2008.01.002 18342479 PMC4345250

[B35] Busquets-Garcia A. Gomis-Gonzalez M. Srivastava R. K. Cutando L. Ortega-Alvaro A. Ruehle S.. (2016). Peripheral and central CB1 cannabinoid receptors control stress-induced impairment of memory consolidation. Proc. Natl. Acad. Sci. U S A 113, 9904–9909. 10.1073/pnas.1525066113 27528659 PMC5024630

[B36] Cachope R. (2012). Functional diversity on synaptic plasticity mediated by endocannabinoids. Philos. Trans. R. Soc. Lond. B Biol. Sci. 367, 3242–3253. 10.1098/rstb.2011.0386 23108543 PMC3481528

[B37] Calik M. W. Radulovacki M. Carley D. W. (2014). Intranodose ganglion injections of dronabinol attenuate serotonin-induced apnea in Sprague-Dawley rat. Respir. Physiol. Neurobiol. 190, 20–24. 10.1016/j.resp.2013.10.001 24121138 PMC3880550

[B38] Campbell S. S. Tobler I. (1984). Animal sleep: a review of sleep duration across phylogeny. Neurosci. Biobehav. Rev. 8, 269–300. 10.1016/0149-7634(84)90054-x 6504414

[B39] Carley D. W. Paviovic S. Janelidze M. Radulovacki M. (2002). Functional role for cannabinoids in respiratory stability during sleep. Sleep 25, 391–398. 10.1093/sleep/25.4.396 12071539

[B40] Castillo P. E. Younts T. J. Chavez A. E. Hashimotodani Y. (2012). Endocannabinoid signaling and synaptic function. Neuron 76, 70–81. 10.1016/j.neuron.2012.09.020 23040807 PMC3517813

[B41] Chemelli R. M. Willie J. T. Sinton C. M. Elmquist J. K. Scammell T. Lee C.. (1999). Narcolepsy in orexin knockout mice: molecular genetics of sleep regulation. Cell 98, 437–451. 10481909 10.1016/s0092-8674(00)81973-x

[B42] Chen R. Zhang J. Fan N. Teng Z. Q. Wu Y. Yang H.. (2013). Δ9-THC-caused synaptic and memory impairments are mediated through COX-2 signaling. Cell 155, 1154–1165. 10.1016/j.cell.2013.10.042 24267894 PMC3918429

[B43] Chesher G. B. Jackson D. M. Starmer G. A. (1974). Interaction of cannabis and general anaesthetic agents in mice. Br. J. Pharmacol. 50, 593–599. 10.1111/j.1476-5381.1974.tb08594.x 4280927 PMC1776719

[B44] Chevaleyre V. Heifets B. D. Kaeser P. S. Sudhof T. C. Castillo P. E. (2007). Endocannabinoid-mediated long-term plasticity requires cAMP/PKA signaling and RIM1α. Neuron 54, 801–812. 10.1016/j.neuron.2007.05.020 17553427 PMC2001295

[B45] Clendinning J. (1843). Observations on the medicinal properties of the Cannabis Sativa of India. Med. Chir. Trans. 26, 188–210. 10.1177/095952874302600116 20895771 PMC2116906

[B142] Colten H. R. Altevogt B. M. Institute of Medicine (US) Committee on Sleep Medicine and Research (Eds.). (2006). Sleep Disorders and Sleep Deprivation: An Unmet Public Health Problem. Wasington, DC: National Academies Press. 20669438

[B46] Copersino M. L. Boyd S. J. Tashkin D. P. Huestis M. A. Heishman S. J. Dermand J. C.. (2006). Cannabis withdrawal among non-treatment-seeking adult cannabis users. Am. J. Addict. 15, 8–14. 10.1080/10550490500418997 16449088

[B47] Corroon J. Kight R. (2018). Regulatory status of cannabidiol in the United States: a perspective. Cannabis Cannabinoid Res. 3, 190–194. 10.1089/can.2018.0030 30283822 PMC6154432

[B48] Cousens K. DiMascio A. (1973). (-)δ9 THC as an hypnotic. An experimental study of three dose levels. Psychopharmacologia 33, 355–364. 10.1007/bf00437513 4776660

[B49] Davis M. I. Ronesi J. Lovinger D. M. (2003). A predominant role for inhibition of the adenylate cyclase/protein kinase A pathway in ERK activation by cannabinoid receptor 1 in N1E-115 neuroblastoma cells. J. Biol. Chem. 278, 48973–48980. 10.1074/jbc.m305697200 14517212

[B50] De Gennaro L. Ferrara M. (2003). Sleep spindles: an overview. Sleep Med. Rev. 7, 423–440. 10.1053/smrv.2002.0252 14573378

[B51] De Oliveira Alvares L. Genro B. P. Diehl F. Quillfeldt J. A. (2008). Differential role of the hippocampal endocannabinoid system in the memory consolidation and retrieval mechanisms. Neurobiol. Learn. Mem. 90, 1–9. 10.1016/j.nlm.2008.01.009 18342551

[B52] Doble A. (1999). New insights into the mechanism of action of hypnotics. J. Psychopharmacol. 13, S11–S20. 10.1177/026988119901304s03 10667451

[B53] Dudok B. Barna L. Ledri M. Szabo S. I. Szabadits E. Pinter B.. (2015). Cell-specific STORM super-resolution imaging reveals nanoscale organization of cannabinoid signaling. Nat. Neurosci. 18, 75–86. 10.1038/nn.3892 25485758 PMC4281300

[B55] Feinberg I. Jones R. Walker J. Cavness C. Floyd T. (1976). Effects of marijuana extract and tetrahydrocannabinol on electroencephalographic sleep patterns. Clin. Pharmacol. Ther. 19, 782–794. 10.1002/cpt1976196782 178475

[B54] Feinberg I. Jones R. Walker J. M. Cavness C. March J. (1975). Effects of high dosage δ-9-tetrahydrocannabinol on sleep patterns in man. Clin. Pharmacol. Ther. 17, 458–466. 10.1002/cpt1975174458 164314

[B56] Filipek S. Palczewski K. Trzaskowski B. Modzelewska A. Latek D. (2012). G protein-coupled receptors—recent advances. Acta Biochim. Pol. 59, 515–529. 23251911 PMC4322417

[B57] Fishbein M. Gov S. Assaf F. Gafni M. Keren O. Sarne Y. (2012). Long-term behavioral and biochemical effects of an ultra-low dose of Δ9-tetrahydrocannabinol (THC): neuroprotection and ERK signaling. Exp. Brain Res. 221, 437–448. 10.1007/s00221-012-3186-5 22821081

[B58] Freemon F. R. (1982). The effect of chronically administered delta-9-tetrahydrocannabinol upon the polygraphically monitored sleep of normal volunteers. Drug Alcohol Depend. 10, 345–353. 10.1016/0376-8716(82)90036-9 6299682

[B59] Friedman E. Gershon S. (1974). Effect of δ8-THC on alcohol-induced sleeping time in the rat. Psychopharmacologia 39, 193–198. 10.1007/bf00421026 4427987

[B60] Garriott J. C. King L. J. Forney R. B. Hughes F. W. (1967). Effects of some tetrahydrocannabinols on hexobarbital sleeping time and amp amphetamine induced hyperactivity in mice. Life Sci. 6, 2119–2128. 10.1016/0024-3205(67)90232-9 6060268

[B61] Garzon J. de la Torre-Madrid E. Rodriguez-Munoz M. Vicente-Sanchez A. Sanchez-Blazquez P. (2009). Gz mediates the long-lasting desensitization of brain CB1 receptors and is essential for cross-tolerance with morphine. Mol. Pain 5:11. 10.1186/1744-8069-5-11 19284549 PMC2657119

[B62] Gates P. Albertella L. Copeland J. (2016). Cannabis withdrawal and sleep: a systematic review of human studies. Subst. Abus. 37, 255–269. 10.1080/08897077.2015.1023484 25893849

[B63] Gorelick D. A. Goodwin R. S. Schwilke E. Schroeder J. R. Schwope D. M. Kelly D. L.. (2013). Around-the-clock oral THC effects on sleep in male chronic daily cannabis smokers. Am. J. Addict. 22, 510–514. 10.1111/j.1521-0391.2013.12003.x 23952899 PMC4537525

[B64] Grant I. Cahn B. R. (2005). Cannabis and endocannabinoid modulators: Therapeutic promises and challenges. Clin. Neurosci. Res. 5, 185–199. 10.1016/j.cnr.2005.08.015 18806886 PMC2544377

[B65] Gremel C. M. Chancey J. H. Atwood B. K. Luo G. Neve R. Ramakrishnan C.. (2016). Endocannabinoid modulation of orbitostriatal circuits gates habit formation. Neuron 90, 1312–1324. 10.1016/j.neuron.2016.04.043 27238866 PMC4911264

[B66] Grundmann M. Merten N. Malfacini D. Inoue A. Preis P. Simon K.. (2018). Lack of beta-arrestin signaling in the absence of active G proteins. Nat. Commun. 9:341. 10.1038/s41467-017-02661-3 29362459 PMC5780443

[B67] Hablitz L. M. Gunesch A. N. Cravetchi O. Moldavan M. Allen C. N. (2020). Cannabinoid signaling recruits astrocytes to modulate presynaptic function in the suprachiasmatic nucleus. eNeuro 7:ENEURO.0081-19.2020. 10.1523/eneuro.0081-19.2020 31964686 PMC7029187

[B68] Hajkova A. Techlovska S. Dvorakova M. Chambers J. N. Kumpost J. Hubalkova P.. (2016). SGIP1 alters internalization and modulates signaling of activated cannabinoid receptor 1 in a biased manner. Neuropharmacology 107, 201–214. 10.1016/j.neuropharm.2016.03.008 26970018

[B69] Halikas J. A. Weller R. A. Morse C. L. Hoffmann R. G. (1985). A longitudinal study of marijuana effects. Int. J. Addict. 20, 701–711. 10.3109/10826088509044290 4044081

[B70] Hammer H. Bader B. M. Ehnert C. Bundgaard C. Bunch L. Hoestgaard-Jensen K.. (2015). A multifaceted GABAA receptor modulator: functional properties and mechanism of action of the sedative-hypnotic and recreational drug methaqualone (quaalude). Mol. Pharmacol. 88, 401–420. 10.1124/mol.115.099291 26056160 PMC4518083

[B71] Han J. Kesner P. Metna-Laurent M. Duan T. Xu L. Georges F.. (2012). Acute cannabinoids impair working memory through astroglial CB1 receptor modulation of hippocampal LTD. Cell 148, 1039–1050. 10.1016/j.cell.2012.01.037 22385967

[B72] Haney M. Ward A. S. Comer S. D. Foltin R. W. Fischman M. W. (1999a). Abstinence symptoms following oral THC administration to humans. Psychopharmacology (Berl) 141, 385–394. 10.1007/s002130050848 10090646

[B73] Haney M. Ward A. S. Comer S. D. Foltin R. W. Fischman M. W. (1999b). Abstinence symptoms following smoked marijuana in humans. Psychopharmacology 141, 395–404. 10.1007/s002130050849 10090647

[B74] Havekes R. Vecsey C. G. Abel T. (2012). The impact of sleep deprivation on neuronal and glial signaling pathways important for memory and synaptic plasticity. Cell. Signal. 24, 1251–1260. 10.1016/j.cellsig.2012.02.010 22570866 PMC3622220

[B75] Hodges E. L. Ashpole N. M. (2019). Aging circadian rhythms and cannabinoids. Neurobiol. Aging 79, 110–118. 10.1016/j.neurobiolaging.2019.03.008 31035036 PMC6591053

[B77] Hoffman A. F. Oz M. Caulder T. Lupica C. R. (2003). Functional tolerance and blockade of long-term depression at synapses in the nucleus accumbens after chronic cannabinoid exposure. J. Neurosci. 23, 4815–4820. 10.1523/jneurosci.23-12-04815.2003 12832502 PMC6741179

[B76] Hoffman A. F. Oz M. Yang R. Lichtman A. H. Lupica C. R. (2007). Opposing actions of chronic Δ9-tetrahydrocannabinol and cannabinoid antagonists on hippocampal long-term potentiation. Learn. Mem. 14, 63–74. 10.1101/lm.439007 17202425 PMC1828281

[B78] Horne J. (2013). Why REM sleep? Clues beyond the laboratory in a more challenging world. Biol. Psychol. 92, 152–168. 10.1016/j.biopsycho.2012.10.010 23174692

[B79] Hsiao Y. T. Yi P. L. Li C. L. Chang F. C. (2012). Effect of cannabidiol on sleep disruption induced by the repeated combination tests consisting of open field and elevated plus-maze in rats. Neuropharmacology 62, 373–384. 10.1016/j.neuropharm.2011.08.013 21867717

[B80] Huitron-Resendiz S. Sanchez-Alavez M. Wills D. N. Cravatt B. F. Henriksen S. J. (2004). Characterization of the sleep-wake patterns in mice lacking fatty acid amide hydrolase. Sleep 27, 857–865. 10.1093/sleep/27.5.857 15453543

[B81] Iber C. Ancoli-Israel S. Chesson A. Quan S. (2007). The AASM Manual for the Scoring of Sleep and Associated Events: Rules, Terminology and Technical Specifications. Westchester, IL: American Academy of Sleep Medicine.

[B82] Kalmbach D. A. Pillai V. Arnedt J. T. Drake C. L. (2016). DSM-5 insomnia and short sleep: comorbidity landscape and racial disparities. Sleep 39, 2101–2111. 10.5665/sleep.6306 27634805 PMC5103798

[B83] Kano M. Ohno-Shosaku T. Hashimotodani Y. Uchigashima M. Watanabe M. (2009). Endocannabinoid-mediated control of synaptic transmission. Physiol. Rev. 89, 309–380. 10.1152/physrev.00019.2008 19126760

[B84] Karacan I. Fernandez-Salas A. Coggins W. J. Carter W. E. Williams R. L. Thornby J. I.. (1976). Sleep electroencephalographic-electrooculographic characteristics of chronic marijuana users: part I. Ann. N Y Acad. Sci. 282, 348–374. 10.1111/j.1749-6632.1976.tb49909.x 190937

[B85] Kimura T. Takaya M. Usami N. Watanabe K. Yamamoto I. (2019). (9)-Tetrahydrocannabinol, a major marijuana component, enhances the anesthetic effect of pentobarbital through the CB1 receptor. Forensic Toxicol. 37, 207–214. 10.1007/s11419-018-0457-2 30636988 PMC6314990

[B86] Koethe D. Schreiber D. Giuffrida A. Mauss C. Faulhaber J. Heydenreich B.. (2009). Sleep deprivation increases oleoylethanolamide in human cerebrospinal fluid. J. Neural Transm. 116, 301–305. 10.1007/s00702-008-0169-6 19137236 PMC2757605

[B87] Kubena R. K. Barry H. III. (1970). Interactions of δ-tetrahydrocannabinol with barbiturates and methamphetamine. J. Pharmacol. Exp. Ther. 173, 94–100. 5442307

[B88] Kuhathasan N. Dufort A. MacKillop J. Gottschalk R. Minuzzi L. Frey B. N. (2019). The use of cannabinoids for sleep: a critical review on clinical trials. Exp. Clin. Psychopharmacol. 27, 383–401. 10.1037/pha0000285 31120284

[B89] Lena I. Parrot S. Deschaux O. Muffat-Joly S. Sauvinet V. Renaud B.. (2005). Variations in extracellular levels of dopamine, noradrenaline, glutamate and aspartate across the sleep–wake cycle in the medial prefrontal cortex and nucleus accumbens of freely moving rats. J. Neurosci. Res 81, 891–899. 10.1002/jnr.20602 16041801

[B90] Levin K. H. Copersino M. L. Heishman S. J. Liu F. Kelly D. L. Boggs D. L.. (2010). Cannabis withdrawal symptoms in non-treatment-seeking adult cannabis smokers. Drug Alcohol Depend. 111, 120–127. 10.1016/j.drugalcdep.2010.04.010 20510550 PMC2930056

[B91] Loflin M. J. Babson K. A. Bonn-Miller M. O. (2017). Cannabinoids as therapeutic for PTSD. Curr. Opin. Psychol. 14, 78–83. 10.1016/j.copsyc.2016.12.001 28813324

[B92] Lonart G. Schoch S. Kaeser P. S. Larkin C. J. Sudhof T. C. Linden D. J. (2003). Phosphorylation of RIM1 α by PKA triggers presynaptic long-term potentiation at cerebellar parallel fiber synapses. Cell 115, 49–60. 10.1016/s0092-8674(03)00727-x 14532002

[B93] Lovinger D. M. (2008). Presynaptic modulation by endocannabinoids. Handb. Exp. Pharmacol. 184, 435–477. 10.1007/978-3-540-74805-2_14 18064422

[B94] Lovinger D. M. Mathur B. N. (2012). Endocannabinoids in striatal plasticity. Parkinsonism Relat. Disord. 18, S132–S134. 10.1016/s1353-8020(11)70415-1 22166411 PMC4791581

[B95] Lu H. C. Mackie K. (2016). An introduction to the endogenous cannabinoid system. Biol. Psychiatry 79, 516–525. 10.1016/j.biopsych.2015.07.028 26698193 PMC4789136

[B96] Lupica C. R. Hoffman A. F. (2018). Cannabinoid disruption of learning mechanisms involved in reward processing. Learn. Mem. 25, 435–445. 10.1101/lm.046748.117 30115765 PMC6097761

[B97] Lynch M. E. Clark A. J. (2003). Cannabis reduces opioid dose in the treatment of chronic non-cancer pain. J. Pain Symptom Manage. 25, 496–498. 10.1016/s0885-3924(03)00142-8 12782429

[B98] Mang G. M. Franken P. (2012). Sleep and EEG phenotyping in mice. Curr. Protoc. Mouse Biol. 2, 55–74. 10.1002/9780470942390.mo110126 26069005

[B99] Marshall L. Born J. (2007). The contribution of sleep to hippocampus-dependent memory consolidation. Trends Cogn. Sci. 11, 442–450. 10.1016/j.tics.2007.09.001 17905642

[B100] Martini L. Thompson D. Kharazia V. Whistler J. L. (2010). Differential regulation of behavioral tolerance to WIN55,212–2 by GASP1. Neuropsychopharmacology 35, 1363–1373. 10.1038/npp.2010.6 20164830 PMC2953419

[B101] Martínez-Vargas M. Murillo-Rodríguez E. González-Rivera R. Landa A. Méndez-Díaz M. Prospro-Garíca O.. (2003). Sleep modulates cannabinoid receptor 1 expression in the pons of rats. Neuroscience 117, 197–201. 10.1016/s0306-4522(02)00820-5 12605905

[B102] Masur J. Khazan N. (1970). Induction by *Cannabis sativa* (marihuana) of rhythmic spike discharges overriding REM sleep electrocorticogram in the rat. Life Sci. I 9, 1275–1280. 10.1016/0024-3205(70)90268-7 4321131

[B103] Mateo Y. Johnson K. A. Covey D. P. Atwood B. K. Wang H. L. Zhang S.. (2017). Endocannabinoid actions on cortical terminals orchestrate local modulation of dopamine release in the nucleus accumbens. Neuron 96, 1112.e5–1126.e5. 10.1016/j.neuron.2017.11.012 29216450 PMC5728656

[B104] Matias I. Vergoni A. V. Petrosino S. Ottani A. Pocai A. Bertolini A.. (2008). Regulation of hypothalamic endocannabinoid levels by neuropeptides and hormones involved in food intake and metabolism: insulin and melanocortins. Neuropharmacology 54, 206–212. 10.1016/j.neuropharm.2007.06.011 17675101

[B105] Mato S. Robbe D. Puente N. Grandes P. Manzoni O. J. (2005). Presynaptic homeostatic plasticity rescues long-term depression after chronic Delta 9-tetrahydrocannabinol exposure. J. Neurosci. 25, 11619–11627. 10.1523/JNEUROSCI.2294-05.2005 16354920 PMC6726043

[B106] Mechoulam R. Fride E. Hanus L. Sheskin T. Bisogno T. Di Marzo V.. (1997). Anandamide may mediate sleep induction. Nature 389, 25–26. 10.1038/37891 9288961

[B107] Mikasova L. Groc L. Choquet D. Manzoni O. J. (2008). Altered surface trafficking of presynaptic cannabinoid type 1 receptor in and out synaptic terminals parallels receptor desensitization. Proc. Natl. Acad. Sci. U S A 105, 18596–18601. 10.1073/pnas.0805959105 19015531 PMC2584146

[B108] Monday H. R. Younts T. J. Castillo P. E. (2018). Long-term plasticity of neurotransmitter release: emerging mechanisms and contributions to brain function and disease. Annu. Rev. Neurosci. 41, 299–322. 10.1146/annurev-neuro-080317-062155 29709205 PMC6238218

[B109] Mondino A. Cavelli M. Gonzalez J. Santana N. Castro-Zaballa S. Mechoso B.. (2019). Acute effect of vaporized Cannabis on sleep and electrocortical activity. Pharmacol. Biochem. Behav. 179, 113–123. 10.1016/j.pbb.2019.02.012 30822492

[B110] Morena M. Roozendaal B. Trezza V. Ratano P. Peloso A. Hauer D.. (2014). Endogenous cannabinoid release within prefrontal-limbic pathways affects memory consolidation of emotional training. Proc. Natl. Acad. Sci. U S A 111, 18333–18338. 10.1073/pnas.1420285111 25489086 PMC4280626

[B111] Mucke M. Weier M. Carter C. Copeland J. Degenhardt L. Cuhls H.. (2018). Systematic review and meta-analysis of cannabinoids in palliative medicine. J. Cachexia Sarcopenia Muscle 9, 220–234. 10.1002/jcsm.12273 29400010 PMC5879974

[B113] Murillo-Rodriguez E. Blanco-Centurion C. Sanchez C. Piomelli D. Shiromani P. J. (2003). Anandamide enhances extracellular levels of adenosine and induces sleep: an *in vivo* microdialysis study. Sleep 26, 943–947. 10.1093/sleep/26.8.943 14746372

[B123] Murillo-Rodriguez E. Cabeza R. Mendez-Diaz M. Navarro L. Prospero-Garcia O. (2001). Anandamide-induced sleep is blocked by SR141716A, a CB1 receptor antagonist and by U73122, a phospholipase C inhibitor. Neuroreport 12, 2131–2136. 10.1097/00001756-200107200-00018 11447321

[B118] Murillo-Rodriguez E. Desarnaud F. Prospero-Garcia O. (2006a). Diurnal variation of arachidonoylethanolamine, palmitoylethanolamide and oleoylethanolamide in the brain of the rat. Life Sci. 79, 30–37. 10.1016/j.lfs.2005.12.028 16434061

[B114] Murillo-Rodriguez E. Millan-Aldaco D. Palomero-Rivero M. Mechoulam R. Drucker-Colin R. (2006b). Cannabidiol, a constituent of Cannabis sativa, modulates sleep in rats. FEBS Lett. 580, 4337–4345. 10.1093/sleep/26.8.943 16844117

[B124] Murillo-Rodriguez E. Machado S. Rocha N. B. Budde H. Yuan T. F. Arias-Carrion O. (2016). Revealing the role of the endocannabinoid system modulators, SR141716A, URB597 and VDM-11, in sleep homeostasis. Neuroscience 339, 433–449. 10.1016/j.neuroscience.2016.10.011 27746343

[B116] Murillo-Rodriguez E. Millan-Aldaco D. Di Marzo V. Drucker-Colin R. (2008a). The anandamide membrane transporter inhibitor, VDM-11, modulates sleep and c-Fos expression in the rat brain. Neuroscience 157, 1–11. 10.1016/j.neuroscience.2008.08.056 18822353

[B115] Murillo-Rodriguez E. Millan-Aldaco D. Palomero-Rivero M. Mechoulam R. Drucker-Colin R. (2008b). The nonpsychoactive Cannabis constituent cannabidiol is a wake-inducing agent. Behav. Neurosci. 122, 1378–1382. 10.1037/a0013278 19045957

[B112] Murillo-Rodriguez E. Sarro-Ramirez A. Sanchez D. Mijangos-Moreno S. Tejeda-Padron A. Poot-Ake A.. (2014). Potential effects of cannabidiol as a wake-promoting agent. Curr. Neuropharmacol. 12, 269–272. 10.2174/1570159x11666131204235805 24851090 PMC4023456

[B117] Murillo-Rodriguez E. Vazquez E. Millan-Aldaco D. Palomero-Rivero M. Drucker-Colin R. (2007). Effects of the fatty acid amide hydrolase inhibitor URB597 on the sleep-wake cycle, c-Fos expression and dopamine levels of the rat. Eur. J. Pharmacol. 562, 82–91. 10.1016/j.ejphar.2007.01.076 17336288

[B120] Murillo-Rodriguez E. Palomero-Rivero M. Millan-Aldaco D. Arias-Carrion O. Drucker-Colin R. (2011a). Administration of URB597, oleoylethanolamide or palmitoylethanolamide increases waking and dopamine in rats. PLoS One 6:e20766. 10.1371/journal.pone.0020766 21779318 PMC3136458

[B121] Murillo-Rodriguez E. Palomero-Rivero M. Millan-Aldaco D. Mechoulam R. Drucker-Colin R. (2011b). Effects on sleep and dopamine levels of microdialysis perfusion of cannabidiol into the lateral hypothalamus of rats. Life Sci. 88, 504–511. 10.1016/j.lfs.2011.01.013 21262236

[B119] Murillo-Rodriguez E. Palomero-Rivero M. Millan-Aldaco D. Di Marzo V. (2013). The administration of endocannabinoid uptake inhibitors OMDM-2 or VDM-11 promotes sleep and decreases extracellular levels of dopamine in rats. Physiol. Behav. 109, 88–95. 10.1016/j.physbeh.2012.11.007 23238438

[B122] Murillo-Rodriguez E. Sanchez-Alavez M. Navarro L. Martinez-Gonzalez D. Drucker-Colin R. Prospero-Garcia O. (1998). Anandamide modulates sleep and memory in rats. Brain Res. 812, 270–274. 10.1016/s0006-8993(98)00969-x 9813364

[B125] Nathan P. J. O’Neill B. V. Napolitano A. Bullmore E. T. (2011). Neuropsychiatric adverse effects of centrally acting antiobesity drugs. CNS Neurosci. Ther. 17, 490–505. 10.1111/j.1755-5949.2010.00172.x 21951371 PMC6493804

[B126] Navarrete M. Araque A. (2008). Endocannabinoids mediate neuron-astrocyte communication. Neuron 57, 883–893. 10.1016/j.neuron.2008.01.029 18367089

[B127] Navarrete M. Araque A. (2010). Endocannabinoids potentiate synaptic transmission through stimulation of astrocytes. Neuron 68, 113–126. 10.1016/j.neuron.2010.08.043 20920795

[B128] Nazzaro C. Greco B. Cerovic M. Baxter P. Rubino T. Trusel M.. (2012). SK channel modulation rescues striatal plasticity and control over habit in cannabinoid tolerance. Nat. Neurosci. 15, 284–293. 10.1038/nn.3022 22231426

[B129] Nguyen P. T. Schmid C. L. Raehal K. M. Selley D. E. Bohn L. M. Sim-Selley L. J. (2012). β-arrestin2 regulates cannabinoid CB1 receptor signaling and adaptation in a central nervous system region-dependent manner. Biol. Psychiatry 71, 714–724. 10.1016/j.biopsych.2011.11.027 22264443 PMC3319102

[B130] Nicholson A. N. Turner C. Stone B. M. Robson P. J. (2004). Effect of Delta-9-tetrahydrocannabinol and cannabidiol on nocturnal sleep and early-morning behavior in young adults. J. Clin. Psychopharmacol. 24, 305–313. 10.1097/01.jcp.0000125688.05091.8f 15118485

[B131] Oishi R. Itoh Y. Nishibori M. Saeki K. Ueki S. (1988). Enhancement by α-fluoromethylhistidine of the thiopental sleep-prolonging action of δ 9-tetrahydrocannabinol. Psychopharmacology 95, 77–81. 10.1007/bf00212771 2838862

[B132] Ong W. Y. Mackie K. (1999). A light and electron microscopic study of the CB1 cannabinoid receptor in primate brain. Neuroscience 92, 1177–1191. 10.1016/s0306-4522(99)00025-1 10426477

[B133] O’Shaughnessy W. B. (1843). On the preparations of the Indian hemp, or Gunjah*- cannabis indica their effects on the animal system in health and their utility in the treatment of tetanus and other convulsive diseases. Prov. Med. J. Retrosp. Med. Sci. 5, 363–369. 10.1136/bmj.s1-5.123.363 30161735 PMC5592602

[B134] Paton W. D. Pertwee R. G. (1972). Effect of cannabis and certain of its constituents on pentobarbitone sleeping time and phenazone metabolism. Br. J. Pharmacol. 44, 250–261. 10.1111/j.1476-5381.1972.tb07261.x 4668592 PMC1666020

[B136] Pava M. J. den Hartog C. R. Blanco-Centurion C. Shiromani P. J. Woodward J. J. (2014). Endocannabinoid modulation of cortical up-states and NREM sleep. PLoS One 9:e88672. 10.1371/journal.pone.0088672 24520411 PMC3919802

[B135] Pava M. J. Makriyannis A. Lovinger D. M. (2016). Endocannabinoid signaling regulates sleep stability. PLoS One 11:e0152473. 10.1371/journal.pone.0152473 27031992 PMC4816426

[B138] Perez-Morales M. De La Herran-Arita A. K. Mendez-Diaz M. Ruiz-Contreras A. E. Drucker-Colin R. Prospero-Garcia O. (2013). 2-AG into the lateral hypothalamus increases REM sleep and cFos expression in melanin concentrating hormone neurons in rats. Pharmacol. Biochem. Behav. 108, 1–7. 10.1016/j.pbb.2013.04.006 23603032

[B137] Perez-Morales M. Fajardo-Valdez A. Mendez-Diaz M. Ruiz-Contreras A. E. Prospero-Garcia O. (2014). 2-Arachidonoylglycerol into the lateral hypothalamus improves reduced sleep in adult rats subjected to maternal separation. Neuroreport 25, 1437–1441. 10.1097/wnr.0000000000000287 25356522

[B139] Perron R. R. Tyson R. L. Sutherland G. R. (2001). Δ9 -tetrahydrocannabinol increases brain temperature and inverts circadian rhythms. Neuroreport 12, 3791–3794. 10.1097/00001756-200112040-00038 11726796

[B140] Piper B. J. DeKeuster R. M. Beals M. L. Cobb C. M. Burchman C. A. Perkinson L.. (2017). Substitution of medical cannabis for pharmaceutical agents for pain, anxiety and sleep. J. Psychopharmacol. 31, 569–575. 10.1177/0269881117699616 28372506

[B141] Pivik R. T. Zarcone V. Dement W. C. Hollister L. E. (1972). Δ-9-tetrahydrocannabinol and synhexl: effects on human sleep patterns. Clin. Pharmacol. Ther. 13, 426–435. 10.1002/cpt1972133426 4337346

[B143] Pranikoff K. Karacan I. Larson E. A. Williams R. L. Thornby J. I. Hursch C. J. (1973). Effects of marijuana smoking on the sleep EEG. Preliminary studies. JFMA 60, 28–31. 4347054

[B144] Priestley R. Glass M. Kendall D. (2017). Functional selectivity at cannabinoid receptors. Adv. Pharmacol. 80, 207–221. 10.1016/bs.apha.2017.03.005 28826535

[B145] Prospéro-García O. Amancio-Belmont O. Becerril Meléndez A. L. Ruiz-Contreras A. E. Méndez-Díaz M. (2016). Endocannabinoids and sleep. Neurosci. Biobehav. Rev. 71, 671–679. 10.1016/j.neubiorev.2016.10.005 27756691

[B146] Rajagopal S. Rajagopal K. Lefkowitz R. J. (2010). Teaching old receptors new tricks: biasing seven-transmembrane receptors. Nat. Rev. Drug Discov. 9, 373–386. 10.1038/nrd3024 20431569 PMC2902265

[B147] Rating D. Broermann I. Honecker H. Kluwe S. Coper H. (1972). Effect of subchronic treatment with (−) 8 -trans-tetrahydrocannabinol (8 -THC) on food intake, body temperature, hexobarbital sleeping time and hexobarbital elimination in rats. Psychopharmacologia 27, 349–357. 10.1007/bf00429388 4648618

[B148] Riedel G. Davies S. N. (2005). “Cannabinoid function in learning, memory and plasticity,” in Cannabinoids, ed Pertwee R. G. (Berlin, Heidelberg: Springer), 445–477. 10.1007/3-540-26573-2_15 16596784

[B149] Romero J. Garcia-Palomero E. Castro J. G. Garcia-Gil L. Ramos J. A. Fernandez-Ruiz J. J. (1997). Effects of chronic exposure to DeltaM^9^-tetrahydrocannabinol on cannabinoid receptor binding and mRNA levels in several rat brain regions. Mol. Brain Res. 46, 100–108. 10.1016/s0169-328x(96)00277-x 9191083

[B152] Rubino T. Forlani G. Vigano D. Zippel R. Parolaro D. (2004). Modulation of extracellular signal-regulated kinases cascade by chronic δ 9-tetrahydrocannabinol treatment. Mol. Cell. Neurosci. 25, 355–362. 10.1016/j.mcn.2003.11.003 15033164

[B151] Rubino T. Vigano D. Massi P. Spinello M. Zagato E. Giagnoni G.. (2000). Chronic Delta-9-tetrahydrocannabinol treatment increases cAMP levels and cAMP-dependent protein kinase activity in some rat brain regions. Neuropharmacology 39, 1331–1336. 10.1016/s0028-3908(99)00196-3 10760375

[B150] Rubino T. Vigano D. Premoli F. Castiglioni C. Bianchessi S. Zippel R.. (2006). Changes in the expression of G protein-coupled receptor kinases and β-arrestins in mouse brain during cannabinoid tolerance: a role for RAS-ERK cascade. Mol. Neurobiol. 33, 199–213. 10.1385/mn:33:3:199 16954596

[B153] Rueda-Orozco P. E. Soria-Gomez E. Montes-Rodriguez C. J. Perez-Morales M. Prospero-Garcia O. (2010). Intrahippocampal administration of anandamide increases REM sleep. Neurosci. Lett. 473, 158–162. 10.1016/j.neulet.2010.02.044 20188142

[B154] Russo E. B. Guy G. W. Robson P. J. (2007). Cannabis, pain, and sleep: lessons from therapeutic clinical trials of Sativex, a cannabis-based medicine. Chem. Biodivers. 4, 1729–1743. 10.1002/cbdv.200790150 17712817

[B155] Sanford A. E. Castillo E. Gannon R. L. (2008). Cannabinoids and hamster circadian activity rhythms. Brain Res. 1222, 141–148. 10.1016/j.brainres.2008.05.048 18582849

[B156] Sanger D. J. (2004). The pharmacology and mechanisms of action of new generation, non-benzodiazepine hypnotic agents. CNS Drugs 18, 9–15; discussion 41, 43–15. 10.2165/00023210-200418001-00004 15291009

[B157] Santucci V. Storme J. J. Soubrie P. LeFur G. (1996). Arousal-enhancing properties of the CB1 cannabinoid receptor antagonist sr 141716A in rats as assessed by electroencephalographic spectral and sleep-waking cycle analysis. Life Sci. 58, Pl103–Pl110. 10.1016/0024-3205(95)02319-4 8569415

[B158] Scammell T. E. Arrigoni E. Lipton J. O. (2017). Neural circuitry of wakefulness and sleep. Neuron 93, 747–765. 10.1016/j.neuron.2017.01.014 28231463 PMC5325713

[B159] Schwartz M. D. Kilduff T. S. (2015). The neurobiology of sleep and wakefulness. Psychiatr. Clin. North Am. 38, 615–644. 10.1016/j.psc.2015.07.002 26600100 PMC4660253

[B160] Shannon S. Lewis N. Lee H. Hughes S. (2019). Cannabidiol in anxiety and sleep: a large case series. Perm. J. 23:18–041. 10.7812/tpp/18-041 30624194 PMC6326553

[B161] Siemens A. J. Kalant H. Khanna J. M. Marshman J. Ho G. (1974). Effect of cannabis on pentobarbital-induced sleeping time and pentobarbital metabolism in the rat. Biochem. Pharmacol. 23, 477–488. 10.1016/0006-2952(74)90612-1 4822738

[B162] Silvani A. Berteotti C. Bastianini S. Lo Martire V. Mazza R. Pagotto U.. (2014). Multiple sleep alterations in mice lacking cannabinoid type 1 receptors. PLoS One 9:e89432. 10.1371/journal.pone.0089432 24586776 PMC3930731

[B163] Smith C. M. (1977). “The pharmacology of sedative/hypnotics, alcohol and anesthetics: sites and mechanisms of action,” in Drug Addiction I, Vol. 45, ed. Martin W. R. (Berlin, Heidelberg: Springer), 413–587.

[B164] Smith T. H. Blume L. C. Straiker A. Cox J. O. David B. G. McVoy J. R.. (2015). Cannabinoid receptor-interacting protein 1a modulates CB1 receptor signaling and regulation. Mol. Pharmacol. 87, 747–765. 10.1124/mol.114.096495 25657338 PMC4366794

[B165] Spindle T. R. Cone E. J. Goffi E. Weerts E. M. Mitchell J. M. Winecker R. E.. (2020). Pharmacodynamic effects of vaporized and oral cannabidiol (CBD) and vaporized CBD-dominant cannabis in infrequent cannabis users. Drug Alcohol Depend. 211:107937. 10.1016/j.drugalcdep.2020.107937 32247649 PMC7414803

[B166] Staner L. (2010). Comorbidity of insomnia and depression. Sleep Med. Rev. 14, 35–46. 10.1016/j.smrv.2009.09.003 19939713

[B167] Steinberg B. A. Cannon C. P. (2007). Cannabinoid-1 receptor blockade in cardiometabolic risk reduction: safety, tolerability and therapeutic potential. Am. J. Cardiol. 100, 27P–32P. 10.1016/j.amjcard.2007.10.011 18154743

[B168] Stella N. (2010). Cannabinoid and cannabinoid-like receptors in microglia, astrocytes, and astrocytomas. Glia 58, 1017–1030. 10.1002/glia.20983 20468046 PMC2919281

[B169] Steriade M. McCarley R. W. (1990). “REM sleep as a biological rhythm,” in Brainstem Control of Wakefulness and Sleep, eds Steriade M. McCarley R. W. (Boston, MA: Springer), 363–393.

[B170] Suraev A. Grunstein R. R. Marshall N. S. D’Rozario A. L. Gordon C. J. Bartlett D. J.. (2020). Cannabidiol (CBD) and Delta^9^-tetrahydrocannabinol (THC) for chronic insomnia disorder (‘CANSLEEP’ trial): protocol for a randomised, placebo-controlled, double-blinded, proof-of-concept trial. BMJ Open 10:e034421. 10.1136/bmjopen-2019-034421 32430450 PMC7239553

[B171] Takata Y. Oishi Y. Zhou X. Z. Hasegawa E. Takahashi K. Cherasse Y.. (2018). Sleep and wakefulness are controlled by ventral medial midbrain/Pons GABAergic neurons in mice. J. Neurosci. 38, 10080–10092. 10.1523/JNEUROSCI.0598-18.2018 30282729 PMC6596202

[B172] Tappe-Theodor A. Agarwal N. Katona I. Rubino T. Martini L. Swiercz J.. (2007). A molecular basis of analgesic tolerance to cannabinoids. J. Neurosci. 27, 4165–4177. 10.1523/jneurosci.5648-06.2007 17428994 PMC6672554

[B173] Tonini R. Ciardo S. Cerovic M. Rubino T. Parolaro D. Mazzanti M.. (2006). ERK-dependent modulation of cerebellar synaptic plasticity after chronic Delta9-tetrahydrocannabinol exposure. J. Neurosci. 26, 5810–5818. 10.1523/jneurosci.5469-05.2006 16723539 PMC6675260

[B174] Toth L. A. Bhargava P. (2013). Animal models of sleep disorders. Comp. Med. 63, 91–104. 10.1007/978-981-10-5981-0_12 23582416 PMC3625050

[B175] Tzavara E. T. Valjent E. Firmo C. Mas M. Beslot F. Defer N.. (2000). Cannabinoid withdrawal is dependent upon PKA activation in the cerebellum. Eur. J. Neurosci. 12, 1038–1046. 10.1046/j.1460-9568.2000.00971.x 10762335

[B176] Valenti M. Vigano D. Casico M. G. Rubino T. Steardo L. Parolaro D.. (2004). Differential diurnal variations of anandamide and 2-arachidonoyl-glycerol levels in rat brain. Cell. Mol. Life Sci. 61, 945–950. 10.1007/s00018-003-3453-5 15095014 PMC11138870

[B178] Vandrey R. Babson K. A. Herrmann E. S. Bonn-Miller M. O. (2014). Interactions between disordered sleep, post-traumatic stress disorder and substance use disorders. Int. Rev. Psychiatry 26, 237–247. 10.3109/09540261.2014.901300 24892898 PMC4052373

[B177] Vandrey R. G. Budney A. J. Hughes J. R. Liguori A. (2008). A within-subject comparison of withdrawal symptoms during abstinence from cannabis, tobacco and both substances. Drug Alcohol Depend. 92, 48–54. 10.1016/j.drugalcdep.2007.06.010 17643868 PMC2214670

[B179] Vandrey R. Smith M. T. McCann U. D. Budney A. J. Curran E. M. (2011). Sleep disturbance and the effects of extended-release zolpidem during cannabis withdrawal. Drug Alcohol Depend. 117, 38–44. 10.1016/j.drugalcdep.2011.01.003 21296508 PMC3119729

[B180] Vaughn L. K. Denning G. Stuhr K. L. de Wit H. Hill M. N. Hillard C. J. (2010). Endocannabinoid signalling: has it got rhythm? Br. J. Pharmacol. 160, 530–543. 10.1111/j.1476-5381.2010.00790.x 20590563 PMC2931554

[B181] Vecsey C. G. Baillie G. S. Jaganath D. Havekes R. Daniels A. Wimmer M.. (2009). Sleep deprivation impairs cAMP signalling in the hippocampus. Nature 461, 1122–1125. 10.1038/nature08488 19847264 PMC2783639

[B182] Wallach M. B. Gershon S. (1973). The effects of Δ8-THC on the EEG, reticular multiple unit activity and sleep of cats. Eur. J. Pharmacol. 24, 172–178. 10.1016/0014-2999(73)90068-x 4358207

[B183] Wallich G. C. (1883). Cannabis indica. Br. Med. J. 1:1224. 10.1136/bmj.1.1173.1224 20750654 PMC2372636

[B184] Ware M. A. Fitzcharles M. A. Joseph L. Shir Y. (2010a). The effects of nabilone on sleep in fibromyalgia: results of a randomized controlled trial. Anesth. Analg. 110, 604–610. 10.1213/ANE.0b013e3181c76f70 20007734

[B185] Ware M. A. Wang T. Shapiro S. Robinson A. Ducruet T. Huynh T.. (2010b). Smoked cannabis for chronic neuropathic pain: a randomized controlled trial. CMAJ 182, E694–E701. 10.1503/cmaj.091414 20805210 PMC2950205

[B186] Whitehurst L. N. Fogler K. Hall K. Hartmann M. Dyche J. (2015). The effects of chronic marijuana use on circadian entrainment. Chronobiol. Int. 32, 561–567. 10.3109/07420528.2015.1004078 25801606

[B187] Willinsky M. D. Scotti de Carolis A. Longo V. G. (1973). EEG and behavioral effects of natural, synthetic and biosynthetic cannabinoids. Psychopharmacologia 31, 365–374. 10.1007/bf00421280 4741417

[B188] Wisor J. P. Jiang P. Striz M. O’Hara B. F. (2009). Effects of ramelteon and triazolam in a mouse genetic model of early morning awakenings. Brain Res. 1296, 46–55. 10.1016/j.brainres.2009.07.103 19664610

[B189] Wu C. S. Zhu J. Wager-Miller J. Wang S. O’Leary D. Monory K.. (2010). Requirement of cannabinoid CB(1) receptors in cortical pyramidal neurons for appropriate development of corticothalamic and thalamocortical projections. Eur. J. Neurosci. 32, 693–706. 10.1111/j.1460-9568.2010.07337.x 21050275 PMC2970673

[B190] Yamashita T. Yamanaka A. (2017). Lateral hypothalamic circuits for sleep-wake control. Curr. Opin. Neurobiol. 44, 94–100. 10.1016/j.conb.2017.03.020 28427008

[B191] Yamauchi M. Kimura H. Strohl K. P. (2010). Mouse models of apnea: strain differences in apnea expression and its pharmacologic and genetic modification. Adv. Exp. Med. Biol. 669, 303–307. 10.1007/978-1-4419-5692-7_62 20217371

[B192] Yim T. T. Hong N. S. Ejaredar M. McKenna J. E. McDonald R. J. (2008). Post-training CB1 cannabinoid receptor agonist activation disrupts long-term consolidation of spatial memories in the hippocampus. Neuroscience 151, 929–936. 10.1016/j.neuroscience.2007.08.037 18248907

[B193] Yim Y. Y. Zurawski Z. Hamm H. (2018). GPCR regulation of secretion. Pharmacol. Ther. 192, 124–140. 10.1016/j.pharmthera.2018.07.005 30056056 PMC6263855

[B194] Younts T. J. Monday H. R. Dudok B. Klein M. E. Jordan B. A. Katona I.. (2016). Presynaptic protein synthesis is required for long-term plasticity of GABA release. Neuron 92, 479–492. 10.1016/j.neuron.2016.09.040 27764673 PMC5119541

[B195] Zhuang S.-Y. Kittler J. Grigorenko E. V. Kirby M. T. Sim L. J. Hampson R. E.. (1998). Effects of long-term exposure to Δ9-THC on expression of cannabinoid receptor (CB1) mRNA in different rat brain regions. Mol. Brain Res. 62, 141–149. 10.1016/s0169-328x(98)00232-0 9813289

[B196] Zimmer A. Zimmer A. M. Hohmann A. G. Herkenham M. Bonner T. I. (1999). Increased mortality, hypoactivity and hypoalgesia in cannabinoid CB1 receptor knockout mice. Proc. Natl. Acad. Sci. U S A 96, 5780–5785. 10.1073/pnas.96.10.5780 10318961 PMC21937

[B197] Zimmerman J. E. Naidoo N. Raizen D. M. Pack A. I. (2008). Conservation of sleep: insights from non-mammalian model systems. Trends Neurosci. 31, 371–376. 10.1016/j.tins.2008.05.001 18538867 PMC2930986

